# Efficacy and Safety of Live Attenuated COVID-19 Vaccine Candidate Based on Omicron-like Temperature-Sensitive Mutant in Preclinical Study

**DOI:** 10.3390/vaccines14100895

**Published:** 2026-10-13

**Authors:** Evgeny Faizuloev, Anastasiia Gracheva, Ekaterina Korchevaya, Daria Khokhlova, Asya Maslennikova, Daria Smirnova, Alina Stog, Daria Nagibina, Galina Trunova, Varvara Khokhlova, Artem Rtishchev, Larisa Kordyukova, Tatiana Trifonova, Andrey Moiseenko, Oxana Svitich, Vitaly Zverev

**Affiliations:** 1 I.I. Mechnikov Research Institute of Vaccines and Sera, 105064 Moscow, Russia; 2 P.A. Hertsen Moscow Oncology Research Institute, 125284 Moscow, Russia; 3 A.N. Belozersky Institute of Physico-Chemical Biology, Lomonosov Moscow State University, 119991 Moscow, Russia; 4 Faculty of Biology, Lomonosov Moscow State University, 119234 Moscow, Russia; 5 F.F. Erisman Institute of Public Health, I.M. Sechenov First Moscow State Medical University ( Sechenov University), 119991 Moscow, Russia

**Keywords:** COVID-19 live attenuated vaccine, temperature-sensitive SARS-CoV-2 mutant, COVID-19 Syrian hamster model, immunization efficacy, transmissibility, *ts* phenotype stability

## Abstract

**Background/Objectives**: Although the COVID-19 pandemic has ended, emerging SARS-CoV-2 variants remain a future threat. This study evaluated the safety and efficacy of an intranasal live attenuated vaccine (LAV) prototype based on a temperature-sensitive (*ts*) Omicron-like SARS-CoV-2 mutant in a Syrian hamster COVID-19 model. **Methods**: SARS-CoV-2 Dubrovka (Wuhan-like), Otradnoe (BA.1.1) and FEB2 (BA.5.2) strains and the *ts* F-F3 mutant derived from FEB2 were characterized by electron microscopy, nanopore sequencing, cytopathic effect titration, and RT-PCR. *In vitro* studies used Vero CCL-81 cells; *in vivo* transmissibility and efficacy studies were assessed in female Syrian hamsters. Serum antibody titers were measured by ELISA and neutralization assays. Immunization efficacy was assessed by weight change; viral loads in the lungs, nasal passages, and brain; and histological examination of the lungs at day 4 post-challenge. Safety criteria included evaluation of pathogenicity and transmissibility in hamsters and *ts* phenotype stability during prolonged *in vitro* cultivation. **Results**: The F-F3 strain displayed a stable *ts* phenotype *in vitro*, did not infect hamster lungs or brain, and showed no transmission from immunized to naïve co-housed animals. A single intranasal immunization by F-F3 induced seroconversion, reduced pneumonia severity by 76.2% and 79.5% in two independent experiments (*p* < 0.01), protected the lungs against productive infection while reducing viral load in the nasal passages, and resulted in no detectable viral transmission after homologous BA.5.2 challenge. Following heterologous Wuhan-like challenge, immunization prevented weight loss and significantly reduced viral loads in the lungs, nasal passages, and brain; however, protection against pneumonia was only modest (18.6%; *p* < 0.05). **Conclusions**: Preclinical evaluation of the F-F3-based LAV prototype demonstrated a favorable balance between safety and efficacy. These characteristics support its further consideration as a candidate strain for COVID-19 LAV development.

## 1. Introduction

Members of *Betacoronavirus* genus within *Coronaviridae* family play a significant role in human respiratory pathology. Human coronaviruses HCoV-OC43 (*Betacoronavirus gravedinis*) and HCoV-HKU1 (*Betacoronavirus hongkongense*) contribute substantially to seasonal acute respiratory diseases morbidity, while zoonotic coronaviruses SARS-CoV-1 (*Betacoronavirus pandemicum*) and MERS-CoV (*Betacoronavirus cameli*) have caused outbreaks of severe respiratory diseases with high mortality rates. Between 2020 and 2023, SARS-CoV-2 (*Betacoronavirus pandemicum*) caused the COVID-19 pandemic, which resulted in at least 7 million deaths worldwide “https://data.who.int/dashboards/covid19/deaths (accessed on 6 March 2026)”. Even countries with highly developed healthcare systems sustained severe losses during the pandemic. Thus, according to WHO estimates, the number of deaths from COVID-19 and its sequelae exceeded 1.2 million in the United States (3612 deaths per million) “https://data.who.int/dashboards/covid19/deaths (accessed on 6 March 2026)”. The true global burden of COVID-19 may be considerably higher than that reflected in publicly accessible data. For instance, due to insufficiently developed epidemiological surveillance systems, accurate assessments of the pandemic’s impact in low-income countries remain unavailable [1]. The severe consequences of the pandemic underscore the inadequate preparedness of national healthcare systems for the emergence of such threats.

Due to herd immunity, the incidence of COVID-19 is currently low, allowing it to be considered a common seasonal respiratory infection. However, the emergence of new SARS-CoV-2 variants of concern or the transmission from animals to humans of other SARS-like coronaviruses that escape post-infection and post-vaccination immunity cannot be excluded [2,3]. In addition, in the post-pandemic period, there is a high proportion of COVID-19 cases with severe course and/or complications in people with concomitant diseases (cerebrovascular and cardiovascular diseases, chronic obstructive pulmonary disease, diabetes) [4]. In this regard, the problem of improving the means of COVID-19 specific prevention remains relevant.

One of the most promising approaches to the specific prophylaxis of respiratory viral infections is the use of intranasally administered mucosal vaccines [5,6]. Mucosal vaccines induce the development of antiviral immunity directly at the respiratory tract mucosa, thereby not only conferring protection against disease but also minimizing the likelihood of viral transmission [5,7,8]. In animal COVID-19 models, intranasally administered mucosal vector vaccines based on replication-incompetent or attenuated human and animal viruses have demonstrated high efficacy [9,10,11,12,13]. Of particular interest are live attenuated vaccines (LAV), which are capable of eliciting robust cellular and humoral immune responses at both the local (mucosal) and systemic levels [14,15,16]. A historical example of the effective use of mucosal LAVs based on *ts* mutants is provided by live influenza vaccines, which are capable of conferring cross-protection against diverse antigenic variants of influenza viruses [17,18]. A number of preclinical studies have demonstrated the high efficacy of attenuated SARS-CoV-2 mutants in protecting susceptible laboratory animals against homologous challenge with a virulent viral strain, even following a single intranasal administration [19,20,21,22,23,24,25,26,27,28].

Based on the results of preclinical studies, a high efficacy of COVID-19 LAV in humans can be anticipated. This assumption is supported by observations of the herd immunity development, which has been formed primarily by the global spread of SARS-CoV-2 and naturally acquired infection. Evidence for the protective effect of natural infection is provided by Chemaitelly H. et al. (2022), who demonstrated that primary SARS-CoV-2 infection conferred 97.3% protection against severe disease or death upon reinfection with an Omicron-like virus, irrespective of the virus variant responsible for the primary infection [29]. In several African countries with low COVID-19 vaccination coverage (e.g., the Democratic Republic of the Congo, Chad, Burundi, Niger, and Nigeria) [30], population immunity has developed predominantly through natural SARS-CoV-2 infection, also reducing morbidity, disease severity, and mortality. This is corroborated by a decline in SARS-CoV-2-positive respiratory samples from over 50% at the 2021–2022 peaks to below 3% in the first half of 2026, comparable to the global average [31]. LAV efficacy is based on the same mechanisms involved in the development of adaptive immunity following natural coronavirus infection [8,32,33], which underscores the substantial potential of LAV in the prevention of diseases caused by SARS-CoV-2-related coronaviruses.

The present study aims to identify a candidate vaccine strain for a COVID-19 LAV. Previously, we generated the SARS-CoV-2 Wuhan-like temperature-sensitive (*ts*) D-D2 mutant, which grows at 23 °C, is sensitive to 39 °C, and displays an attenuated (*att*) phenotype in golden Syrian hamsters [28]. A single intranasal immunization with D-D2 reproducibly induced seroconversion in all immunized hamsters and provided highly effective protection against homologous (parental Wuhan-like) and heterologous (Delta- and Omicron-like) challenge [19,28]. However, because this *ts* mutant, derived from the highly virulent neurotropic Dubrovka strain, retained limited replication in hamster lungs and induced focal interstitial pneumonia [28], it cannot be considered entirely safe.

We therefore selected the less virulent Omicron-like (BA.5.2) FEB2 strain for further attenuation. FEB2 induces pneumonia in hamsters but does not cause weight loss or infect the brain [34], and its antigenic relatedness to currently circulating Omicron-like variants further justified this choice. By passaging FEB2 in Vero cells at reduced temperature, we for the first time generated Omicron-like *ts* mutant, F-F3, which grows at 24 °C and is sensitive to 37 °C and above. F-F3 is avirulent for hamsters, having lost the ability to infect the lungs while retaining replication in the nasal passages and immunogenicity [35], positioning it as a candidate LAV strain.

LAV development may be complicated by “hyperattenuation” in which a mutant loses immunogenicity after adaptation to novel conditions, as well as by residual virulence or *att* phenotype instability. A critical limitation of any LAV is the potential transmission of the vaccine strain from immunized individuals to naïve contacts, with subsequent circulation and possible reversion to virulence, as documented for the oral poliovirus vaccine [36]. The most crucial safety characteristics of a vaccine strain are therefore its non-transmissibility and *att* phenotype stability.

The aim of the study was to evaluate the safety and efficacy of a live attenuated vaccine prototype based on an Omicron-like SARS-CoV-2 *ts* mutant in an animal model of coronavirus pneumonia. Syrian hamsters were selected for *in vivo* studies, since the Syrian hamster COVID-19 model is well characterized and broadly accepted as an animal model of the disease.

## 2. Materials and Methods

### 2.1. Viruses

The following SARS-CoV-2 laboratory strains were used in the study: Wuhan-like Dubrovka strain, Omicron-like FEB2 and Otradnoe strains [34], and cold-adapted (*ca*) mutants derived from the FEB2 strain (F-F1, F-F3, F-D3, F-D12, F-C5, and F-C9), which were generated previously in our laboratory [35] (Table 1).

### 2.2. Animals

Four-week-old female golden Syrian hamsters (*Mesocricetus auratus*) weighing 40–50 g were obtained from the Nursery for Laboratory Animals of the Shemyakin and Ovchinnikov Institute of Bioorganic Chemistry of the RAS (Pushchino, Russia). SPF hamsters were screened at the nursery and confirmed free of viral, bacterial, and parasitic infections. Following delivery from the nursery, the animals were acclimatized for 4–7 days prior to the start of the experiments. Hamsters were housed under standard laboratory conditions at a controlled temperature of 20–26 °C and relative humidity of 45–65%, with a 12 h light/12 h dark cycle. Different groups of animals were maintained in individual cages. Standard rodent diet and water were provided *ad libitum*. Animals were randomly allocated to the experimental groups using a computer-generated random number sequence with Microsoft Excel 2013. Group size was dedicated to allow for an assessment of the significance of between-group differences. No a priori inclusion or exclusion criteria were established for this study. All animals and data points were included in the analysis; no exclusions were applied. Hamsters were inoculated intranasally with SARS-CoV-2 in a volume of 100 μL containing 5.0 or 6.0 log_10_ TCID_50_, administered to both nostrils under light anesthesia. Upon termination of the experimental procedures, the hamsters were humanely euthanized by cervical dislocation following anesthesia. Animal suffering during anesthesia and euthanasia was minimized by performing all procedures under the supervision of a qualified veterinarian. The animals were monitored daily throughout the experiment Predefined humane endpoints were established prior to the study and included loss of ≥20% of baseline body weight, severe respiratory distress, and pronounced lethargy. Animals meeting any of these criteria were to be humanely euthanized immediately. No animals reached the predefined humane endpoints before the scheduled time points; nevertheless, these criteria were applied throughout the study. Hamsters were housed in accordance with the European Convention for the Protection of Vertebrate Animals Used for Experimental and Other Scientific Purposes [37], and all procedures complied with the Order of the Ministry of Health of the Russian Federation No. 199n of 1 April 2016, “On Approval of the Rules of Good Laboratory Practice.” All procedures involving animals were approved by the Local Ethics Committee of the I.I. Mechnikov Research Institute of Vaccines and Sera prior to the commencement of the study (Protocol No. 2 dated 24 May 2021).

### 2.3. Cell and Virus Cultivation

Vero CCL-81 (American Type Culture Collection, Manassas, VA, USA) cells were cultured at 37 °C in DMEM medium based on Earle’s buffer (PanEco, Moscow, Russia) supplemented with 5% fetal bovine serum (FBS) (Gibco, Grand Island, NY, USA), 300 μg/mL L-glutamine (PanEco), and 40 μg/mL gentamicin (PanEco) in a 5% CO_2_ atmosphere. SARS-CoV-2 was propagated in Vero cells as previously described [38]. The maintenance medium consisted of DMEM supplemented with 300 μg/mL L-glutamine, 40 μg/mL gentamicin, and 1% FBS. Vero cell monolayers were infected with SARS-CoV-2 and incubated at 37 °C for 3–4 days (strains Dubrovka, Otradnoe, and FEB2, MOI 0.001–0.00001) or at 24 °C for 7–10 days (FEB2 strain *ca* mutants, MOI 0.01) in a 5% CO_2_ atmosphere until pronounced cytopathic effect (CPE) became evident. The virus-containing culture medium was clarified by centrifugation at 4500× *g* for 5 min, aliquoted, titrated, and stored at −80 °C until use.

### 2.4. Virus Titration

SARS-CoV-2 titer was determined by TCID_50_ endpoint dilution assay in Vero CCL-81 cell culture, as previously described [38]. Dubrovka, Otradnoe, and FEB2 strains were titrated at 37 °C, whereas the FEB2 strain *ca* mutants were titrated at 30 °C. The virus titer was calculated according to Ramakrishnan M.A. et al. (2016) [39] and expressed as log_10_ TCID_50_/mL.

### 2.5. Quantification of SARS-CoV-2 RNA

SARS-CoV-2 RNA was quantified by real-time RT-PCR using a DTprime amplification system (DNA-Technology, Moscow, Russia), as described previously [19]. Viral RNA was extracted from samples using the MagnoPrime UNI reagent kit (NextBio, Moscow, Russia). For the detection of viral RNA, primers and a probe targeting SARS-CoV-2 nucleocapsid (N) gene were employed: CoVN-F (GCGTTCTTCGGAATGTCG), CoVN-R (TTGGATCTTTGTCATCCAATTTG), and CoVN-P (FAM-AACGTGGTTGACCTACACAGGT-BHQ1) [40]. A synthetic oligonucleotide corresponding to the amplified fragment of the SARS-CoV-2 genome was used to construct a standard calibration curve.

### 2.6. SARS-CoV-2 Genome Sequencing

SARS-CoV-2 genome sequencing was performed on a MinION nanopore sequencer using Flow Cell R9.4 with the MinKNOW software v. 24.11.10 and reagent kits from Oxford Nanopore Technologies (Oxford, UK), as described previously [28]. Specific RT-PCR amplification of the SARS-CoV-2 genome was carried out using the NEBNext^®^ ARTIC SARS-CoV-2 Companion Kit (NEB, Ipswich, MA, USA). Ligation Sequencing kit and Native Barcoding Kit 1D (Oxford Nanopore Technologies) were used to prepare the resulting pool. Genome assembly was performed in Minimap2 v. 2.24 [41].

### 2.7. Transmission Electron Microscopy Analysis

The virus-containing cell culture medium was clarified at low centrifugation speed (2900× *g* 10 min, 4 °C, A-4-44 rotor, Eppendorf, Hamburg, Germany) and was passed through Amicon Ultra Centrifugal Filters (Millipore, Burlington, MA, USA) with a cut-off of 100,000 Da (100 K) at a low centrifugation speed (3600× *g* 10 min, 4 °C; A-4-44 rotor, Eppendorf). The concentrated virus particles were re-suspended in a sterile phosphate-buffered saline (PBS, pH 7.2), and aliquots of the virus suspension were frozen (−80 °C) until usage. To obtain immune complexes, the virus sample was thawed, mixed with rabbit serum against the FEB2 strain, and incubated for 1 h at 37 °C. The virus samples or immune complexes were loaded onto formvar-coated grids (TedPella, Redding, CA, USA), stained with a 2% water solution of phosphotungstic acid (PTA) (Fluka, Buchs, Switzerland), pH 7.0, and viewed with a transmission electron microscope JEM-2100 (JEOL, Tokyo, Japan). The research was carried out at the Shared Research Facility “Electron microscopy in life sciences” at Moscow State University (Unique Scientific Equipment “Three-Dimensional Electron Microscopy and Spectroscopy”, ID RFMEFI61919X0014).

### 2.8. Detection of Ts Phenotype in Ca SARS-CoV-2 Mutants

Vero cells were infected with parental FEB2 strain and *ca* mutants at MOI 0.001. Following adsorption for 1 h, the cells were washed twice with serum-free medium, supplemented with maintenance medium, and incubated at 37 °C and 39 °C for 4 days in 5% CO_2_ atmosphere. From days 1 through 4 post-infection (p.i.), culture supernatant samples were collected daily, and viral titers as well as viral RNA concentrations were determined. A reduction in infectious titer or viral RNA concentration of 4.0 log or more on day 4 p.i. compared to FEB2 strain was considered indicative of a *ts* phenotype.

### 2.9. Assessment of F-F3 Strain Ts Phenotype Stability

F-F3 strain (propagated at 24 °C) and the parental FEB2 strain (propagated at 37 °C) were serially passaged for 10 passages in Vero cells in 25 cm^2^ culture flasks under two temperature conditions—33 °C and 37 °C. For each subsequent passage, 100 μL of culture supernatant collected on day 4 p.i. were inoculated (without prior virus titration) into flasks containing a 3-day-old cell monolayer and incubated in a 5% CO_2_ atmosphere. At each passage level, the presence of CPE was assessed on day 4 p.i. Viral material harvested after the 10th passage was evaluated for infectious titer and viral RNA concentration. Additionally, viral material obtained after the 10th passage was tested for temperature sensitivity at 37 °C and 39 °C based on viral RNA accumulation, as described above.

### 2.10. Evaluation of Immunization Efficacy

**Figure 1:**
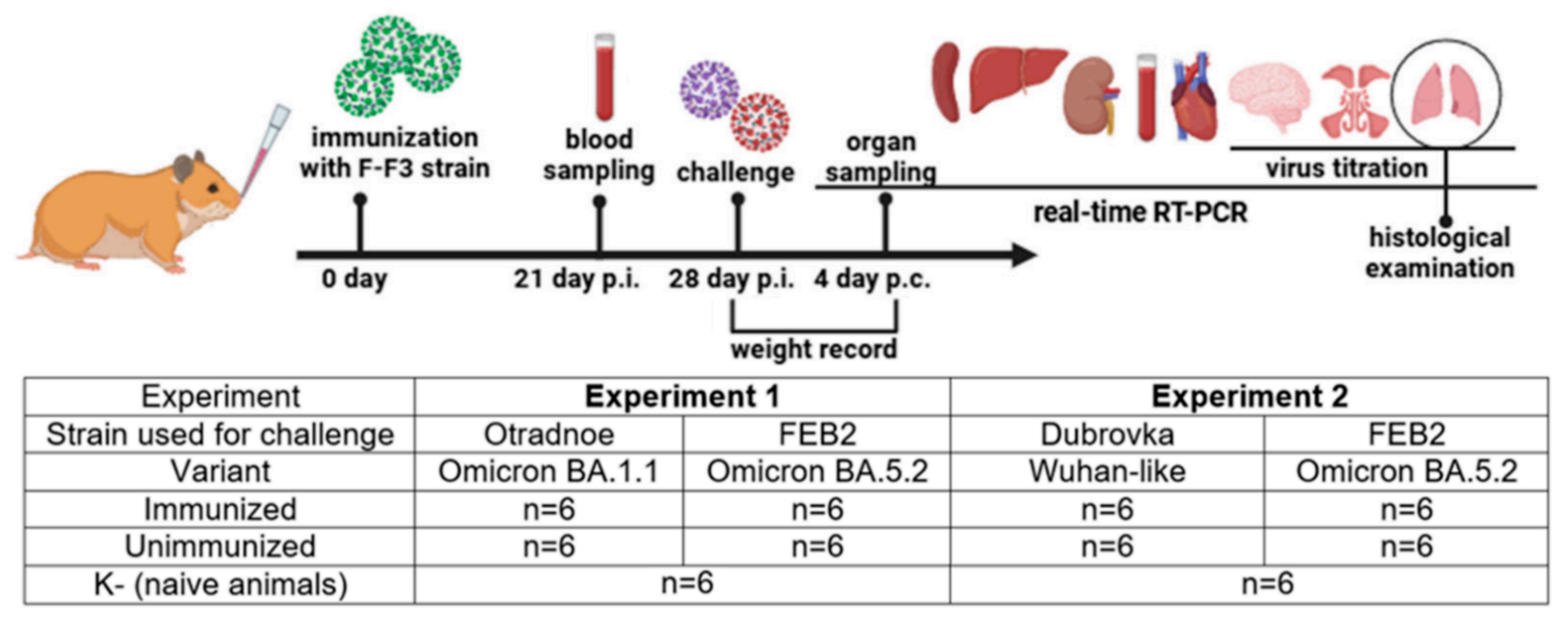
Study design for the evaluation of immunogenicity and efficacy immunization with F-F3 strain. K-—unimmunized unchallenged (naïve) animals. **p.i.**—post-immunization; **p.c.**—post-challenge. The illustration was prepared using BioRender (https://www.biorender.com/).

The efficacy of immunization with F-F3 strain was assessed in two independent experiments according to the experimental schedule (Figure 1). A study protocol, including the research question, key design features, and analysis plan, was prepared prior to the study; however, it was not registered.

For each experiment, hamsters were divided into five groups of six animals each (total number of animals, 60). Hamsters in two experimental groups received 100 μL of F-F3 strain at a dose of 5.0 log_10_ TCID_50_ per animal intranasally. Non-immunized hamsters in two control groups received 100 μL of PBS, pH 7.2, intranasally. At 28 p.i., immunized and non-immunized hamsters in the first experiment were challenged intranasally with FEB2 and Otradnoe SARS-CoV-2 strains, while in the second experiment, they were challenged with FEB2 and Dubrovka strains, at a dose of 5.0 log_10_ TCID_50_ per animal. An additional control group consisted of naïve (non-immunized and non-challenged) hamsters. Animals were monitored daily, and body weight was recorded from day 0 to day 4 post-challenge (p.c.). At 4 days p.c., hamsters were humanely euthanized. The right lung, brain, nasal passages, liver, spleen, heart, and kidneys were excised, rinsed, and homogenized in DMEM supplemented with gentamicin (40 μg/mL) using Bioprep-24R homogenizer (Allsheng, Hangzhou, China) (4000 rpm × 4.5 min, 4 °C). The resulting homogenates were clarified by centrifugation (9500× *g* 5 min, 4 °C). Clarified organ homogenates were stored at −80 °C for subsequent virus titration and viral RNA quantification. The left lung was preserved in 10% neutral buffered formalin for histological examination. Immunization efficacy was assessed by comparing weight dynamics, viral load in organs, and histopathological changes in the lungs between immunized and non-immunized hamsters. To minimize the influence of potential confounders, animals in each group were housed in separate cages, and a separate container was used for weighing each group. In addition, cages containing non-immunized animals were isolated from those containing immunized animals prior to challenge to prevent airborne transmission of the virus. The investigators responsible for animal weighing, RT-PCR, virus titration, histological examination, and outcome assessment were blinded to group allocation.

### 2.11. Evaluation of Protection Against Transmission upon Challenge of Immunized Hamsters with a Virulent Strain

Two groups of hamsters (*n* = 12 and *n* = 4) were immunized intranasally with F-F3 strain at a dose of 5.0 log_10_ TCID_50_ per animal. At 28 days p.i., one group of hamsters was challenged intranasally with Dubrovka strain, while the second group was challenged with FEB2 strain at a dose of 5.0 log_10_ TCID_50_/mL. On the following day, four naïve hamsters were introduced into each cage containing the challenged animals and were co-housed until the end of the experiment. At day 21 p.c., blood samples were collected from all hamsters for the detection of IgG antibodies to SARS-CoV-2 by ELISA and neutralization assay. The absence of seroconversion in the co-housed animals was considered the criterion for protection against transmission.

### 2.12. Assessment of Immunogenicity

F-F3 strain immunogenicity was evaluated in two independent experiments by quantifying total and virus-neutralizing antibodies in hamster sera at 21 days post-immunization (p.i.). Hamsters (*n* = 12) were administered 100 μL of strain F-F3 at a dose of 5.0 log_10_ TCID_50_ per animal intranasally under light anesthesia, according to the experimental schedule (Figure 1). In the first experiment, an additional group of hamsters (*n* = 6) was inoculated with the parental FEB2 strain at an equivalent dose. Control group hamsters (*n* = 6) received 100 μL of PBS, pH 7.2, intranasally (K- group).

### 2.13. Determination of Antibodies to SARS-CoV-2

IgG antibodies to SARS-CoV-2 structural antigens in hamster sera were determined by ELISA, as described previously [28]. For ELISA, 96-well plates were coated with whole-virion preparation of SARS-CoV-2 Dubrovka strain, inactivated by UV irradiation, which was obtained as previously described [42]. The amount of viral antigen per well of ELISA plate corresponded to approximately 5.5 log_10_ TCID_50_. Neutralizing antibodies titer was determined in Vero cell culture against the FEB2 and Dubrovka strains, as described earlier [38].

### 2.14. Histopathology Evaluation of the Lungs

During autopsy of the experimental animals performed on the 4th day p.c. the left lung was removed for further histological investigation. After fixation in 10% buffered formalin for 24 h and standard histological processing, the step sections of the organ were prepared and stained with hematoxylin and eosin. Photographic recording, microscopic examination of lung sections, and semi-quantitative assessment of the severity of histopathological changes were performed blindly by a veterinary pathologist with mandatory preliminary checking of intra-expert agreement. The area of the lesion and the nature of the spatial distribution of pathological changes in the lung were determined at low magnification using an OLYMPUS BX51 microscope (Olympus, Tokyo, Japan) on 3 longitudinally oriented step sections per animal. For this purpose, the sections were examined completely, without using a fixed number of fields of view. The condition of the affected areas was assessed, if necessary, at higher magnification in 10 fields of view of ×10–×63 lenses according to the severity of specific alterative-inflammatory changes using a scale from 0 to 3 points. Then, each encrypted histological preparation was assigned a cumulative severity score, calculated by adding the average scores (from 0 to 3) for each morphological assessment criterion proposed in the work of Gruber A.D. et al. (2020) [43]. The morphological evaluation criteria included the following: % of affected lung area, distribution pattern of pneumonia foci, cellular and tissue damage (necrosis of bronchial and bronchiole epithelium, alveolar cell necrosis, and cellular debris in the alveoli), hemodynamic impairment and vascular damage (intraalveolar hemorrhage, intraalveolar edema, perivascular and interstitial edema, vascular wall inflammation, endothelial necrosis, and desquamation), inflammation (bronchitis, bronchointerstitial pneumonia, interstitial pneumonia, macrophages and neutrophils in the alveolar lumen, perivascular lymphocytic cuffs), regeneration, and repair (hyperplasia of bronchial ciliated epithelium and type II alveolar cell hyperplasia). When forming the total score, the results obtained for one animal for each criterion were summed up, and the average score was determined and rounded to a whole number.

### 2.15. Assessment F-F3 Strain Transmissibility

In two independent experiments, hamsters (*n* = 12) were immunized intranasally with F-F3 strain at a dose of 5.0 log_10_ TCID_50_ per animal. On the day following immunization, naïve hamsters (*n* = 4) were introduced into the cage and co-housed with the immunized animals throughout the entire experiment. A separate cage housed naïve hamsters serving as the control group (*n* = 4). At 21 days p.i., blood samples were collected from all hamsters and tested for IgG antibodies to SARS-CoV-2 by ELISA and neutralization assay. Seroconversion in the co-housed animals was considered the criterion for transmission.

In an additional experiment, 15 naïve hamsters were divided into 5 equal groups of 3 animals each (groups P0, P1, P2, P3, and P4). P0 group was infected intranasally with F-F3 strain at a dose of 6.0 log_10_ TCID_50_ per animal. At 3 days p.i., nasal washes were collected from the infected animals under light anesthesia by flushing the nasal passages with DMEM supplemented with gentamicin (40 μg/mL). The washes were pooled and administered intranasally (50 μL) to P1 group hamsters. This procedure was repeated for a total of 4 sequential passages (groups P1, P2, P3, and P4). All nasal wash samples were analyzed for the presence of viral RNA and infectious virus. At 21 days p.i., blood samples were collected from all hamsters and tested for IgG antibodies to SARS-CoV-2 by ELISA. The criteria for infection of hamsters included the detection of seroconversion, as well as the presence of viral RNA and infectious virus in nasal wash samples.

### 2.16. Statistical Analysis

Each animal was considered an experimental unit, and each group of animals was housed in a separate cage. However, this housing arrangement does not exclude a possible cage effect: because each group was housed in a single cage, treatment and cage effects cannot be fully separated, and this limitation should be taken into account when interpreting the results. The distribution of animals across groups and cages for all experiments involving animals is provided in Supplemental Table S1. Group sizes were selected based on the laboratory’s previous experience with the Syrian hamster SARS-CoV-2 model and the magnitude of between-group differences observed in prior experiments [19,34]. The sample size was also limited in accordance with the principles of reduction in animal experimentation. No formal a priori power calculation was performed.

Due to the small group sizes and the non-normal distribution expected for virological endpoints, between-group comparisons of continuous variables were performed using the two-sided Mann–Whitney U test. Statistical significance was assessed at a two-sided α level of 0.05. Exact *p*-values are reported where appropriate. No adjustment for multiple testing was applied, as the analyses were considered exploratory and involved comparisons across different challenge strains, tissues, and outcome measures. Data are presented as mean ± standard error of the mean (SEM) for body weight measurements or interquartile range (IQR) for single-time-point virological, histopathological, and serological endpoints. Statistical analyses and data visualization were performed using GraphPad Prism software (version 8). For graphical presentation and statistical analysis, values below the limit of detection were assigned their assay-specific values: 50 for the IgG titer, 10 for the neutralizing antibody titer, 3.0 log_10_ RNA copies/mL for RNA detection, and 2.0 log_10_ TCID_50_/mL for the virus titer.

In efficacy experiments, all virological and histopathological endpoints were assessed once, on day 4 p.c., and therefore did not involve repeated measurements within individual animals. Effect sizes are presented as the Hodges–Lehmann median difference between the compared groups of immunized and non-immunized animals, separately for viral load (virus titer and RNA concentration) and pneumonia severity scores, with the *p*-value and 95% CI reported. The protective effect against pneumonia was additionally expressed as the percentage reduction (PR, %):

PR(%) = [(M_controls − M_immunized)/M_controls] × 100,

where M_controls and M_immunized are the median cumulative pneumonia severity scores of the non-immunized control and immunized groups, respectively.

Body weight was recorded repeatedly from day 0 to day 4 p.c. To avoid treating repeated measurements from the same animal as independent observations, body weight was not analyzed as separate daily observations. For statistical comparison, the prespecified endpoint was the change in body weight from day 0 to day 4 p.c., expressed as a percentage of baseline body weight (BW) for each animal:

ΔBW(%) = [(BWday4 − BWday0)/BWday0] × 100.

The resulting individual day-4 change-from-baseline values were compared between immunized and non-immunized animals using the two-sided Mann–Whitney U test.

In the transmission experiments, each cage was considered a single independent transmission event, and the data are presented qualitatively rather than as independent replicates.

### 2.17. Biosafety Requirements

All work with the SARS-CoV-2 virus was carried out under conditions of a Biosafety Level-3 laboratory.

## 3. Results

### 3.1. F-F3 Strain Characterization

Previously, through SARS-CoV-2 FEB2 strain cold adaptation, six *ca* mutant clones capable of replicating in Vero cells at 24 °C were obtained, F-F1, F-F3, F-D3, F-D12, F-C5, and F-C9 (Table 1) [35], and their genomic sequences were determined (GenBank accession numbers PX401966–PX401971). Clones F-C5 and F-C9 exhibited temperature sensitivity at 39 °C but not at 37 °C, whereas clones F-F1, F-F3, F-D3, and F-D12 were sensitive at 37 °C [35]. Comparative analysis of parental FEB2 strain (GenBank accession number OP920753.1) and its *ca* mutants genomes revealed genetic changes that occurred as a result of viral adaptation to growth in Vero cells at 24 °C. The genomes of the *ca* mutants contained 21 to 24 nucleotide substitutions (Supplemental Table S2), predominantly nonsynonymous (Supplemental Table S3). For further investigation, F-F3 *ts* strain (GenBank accession number PX401966.1) was selected, which acquired 23 nucleotide substitutions (Supplemental Table S2), 20 of which resulted in amino acid changes in the NSP3, NSP5, NSP6, NSP12, NSP13, NSP14, S, E, M, and N proteins (Supplemental Table S3). Thus, a set of candidate mutations associated with the *ca*/*ts*/*att* phenotype was identified.

**Figure 2:**
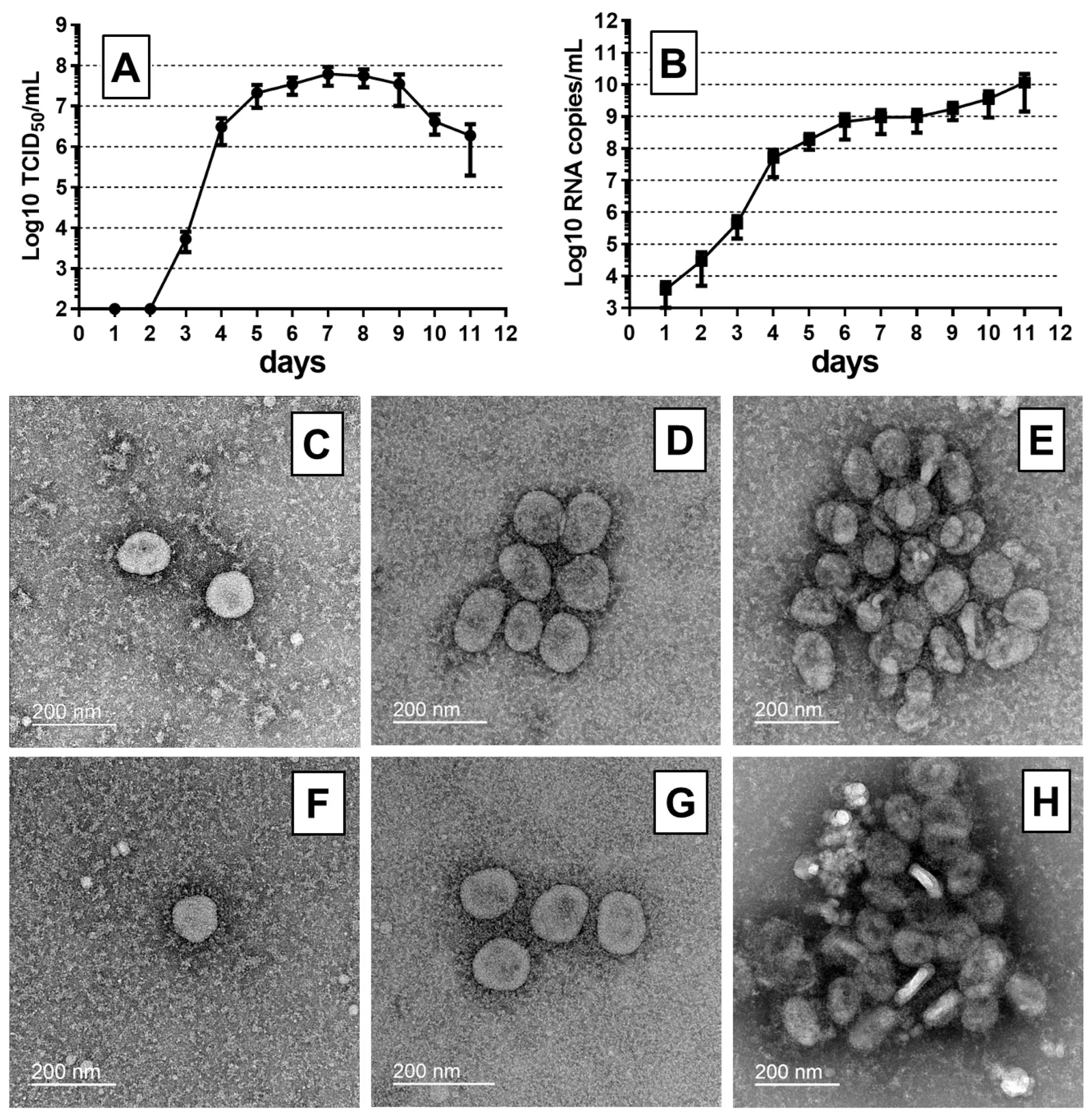
F-F3 strain replication. Accumulation of infectious virus (**A**) and viral RNA (**B**) in culture supernatant of Vero cells infected with F-F3 strain (MOI 0.01) at 24 °C. Data from two independent experiments are shown as mean ± SEM. The transmission electron microscopy images of negatively stained virions of FEB2 (**C**) and F-F3 (**F**) strains, as well as immune complexes of FEB2 (**D**,**E**) and F-F3 (**G**,**H**) strains with immune rabbit serum against FEB2 strain are represented.

In Vero cells infected with the F-F3 strain (MOI 0.01) at 24 °C, infectious virus accumulated in culture supernatant, reaching peak titers of 7.4–7.7 log_10_ TCID_50_/mL on days 6–9 p.i., after which infectious activity gradually declined by day 11 (Figure 2A). The accumulation of infectious virus in culture fluid was corroborated by an increase in viral RNA concentration (Figure 2B). CPE was mild and manifested as alterations in cell morphology. Noticeable detachment of individual virus-infected cells from the flask surface began only after days 6–7 p.i. In preparations of the F-F3 strain propagated at 24 °C, coronavirus virions were observed, which were similar in morphology and size to those of the parental FEB2 strain (cultivated at 37 °C) (Figure 2C–H). Virions from both virus preparations reacted with hyperimmune rabbit serum against FEB2 strain, forming immune complexes of various sizes, indicating that the surface proteins of F-F3 retained the antigenic properties of the parental strain (Figure 2D,E,G,H) [44]. Within the immune complexes, a considerable number of elongated structures were observed in both preparations, comparable in length to the diameter of a coronavirus virion. These elongated structures likely represent defective nucleocapsid-deficient (empty) virions, which become flattened upon drying, assume a disc-like shape, and are oriented perpendicular to the grid surface.

### 3.2. F-F3 Strain Ts Phenotype Stability In Vitro

**Figure 3:**
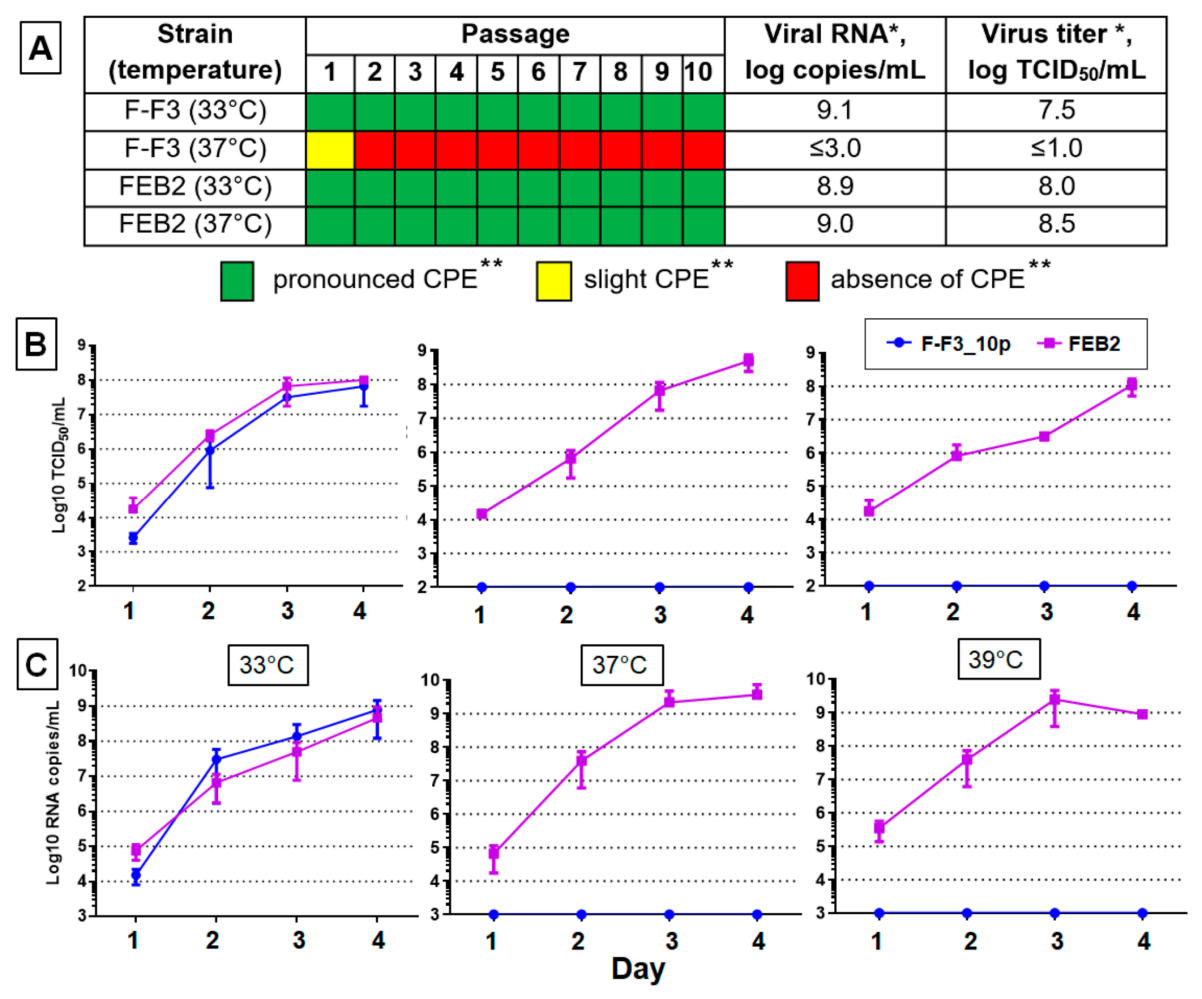
F-F3 strain *ts* phenotype stability *in vitro*. Passaging of F-F3 and FEB2 strains in Vero cells at 33 °C and 37 °C (**A**). * Analysis of culture supernatant obtained after the 10th passage on day 4 p.i. ** CPE manifestation on day 4 p.i. Evaluation of F-F3 strain temperature sensitivity after 10 passages at 33 °C (F-F3_10p) at 37 °C and 39 °C in Vero cell culture (MOI 0.001) (**B**,**C**). FEB2 strain was used as a reference. Kinetics of virus titer (**B**) and viral RNA concentration (**C**) from days 1 to 4 p.i. Data are from two independent experiments. Error bars = SEM.

To assess the possibility of *ts* phenotype reversion (i.e., reversion to the non-*ts* phenotype characteristic of the parental FEB2 strain), F-F3 strain was serially passaged for 10 passages in Vero cell culture under temperatures 33 °C and 37 °C. At 33 °C, F-F3 strain replicated at all passage levels, inducing pronounced CPE by days 3–4 p.i., with both infectious virus and viral RNA detectable in the culture supernatant. At 37 °C, infection of Vero cells with F-F3 strain was abortive, as evidenced by CPE absence from passages 2 through 10, as well as the lack of infectious virus and viral RNA in the culture fluid (Figure 3A). FEB2 reference strain replicated efficiently under both temperature conditions.

After 10 passages at 33 °C, F-F3 strain (F-F3_10p) retained its temperature sensitivity at both 37 °C and 39 °C. At 33 °C, F-F3_10p replicated productively, accompanied by pronounced CPE and increases in virus titer and viral RNA concentration in culture supernatant, whereas at 37 °C and 39 °C, no signs of viral replication were detected. FEB2 reference strain replicated efficiently at all three temperatures (33 °C, 37 °C, and 39 °C) (Figure 3B,C). The obtained results indicate F-F3 strain *ts* phenotype stability under long-term cultivation *in vitro* at 33 °C.

### 3.3. F-F3 Strain Immunogenicity

**Figure 4:**
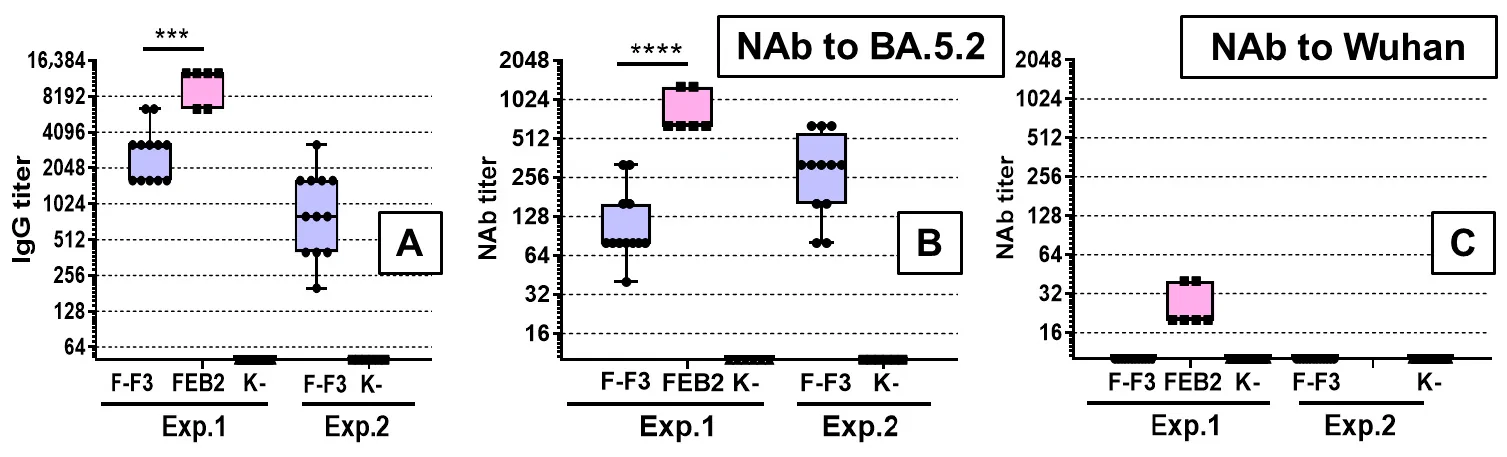
Antibody titers to SARS-CoV-2 in hamster sera at 21 days after a single intranasal immunization. IgG titers to SARS-CoV-2 antigens measured by ELISA (**A**). NAb titers against the homologous BA.5.2-like FEB2 strain (**B**) and the heterologous Wuhan-like Dubrovka strain (**C**). **NAb**—neutralizing antibodies. Data from two independent experiments. **Exp.1**, **Exp.2**—experiments 1 and 2. ***—*p* < 0.001; ****—*p* < 0.0001 (Mann–Whitney U test). Data presented using individual data points and median/IQR.

F-F3 strain immunogenicity was evaluated in two independent experiments according to the scheme (Figure 1). In both experiments, all immunized hamsters developed both IgG antibodies to SARS-CoV-2 antigens and neutralizing antibodies (Nab) in serum at 21 days following a single intranasal immunization. Notably, IgG and Nab titers in hamsters immunized with F-F3 were fourfold (*p* < 0.001; 9600; 95% CI: 4800–11200) and eightfold (*p* < 0.0001; 560; 95% CI: 480–1200) lower, respectively, compared to the group infected with an equivalent dose of the parental FEB2 strain (Figure 4).

### 3.4. F-F3 Strain Immunization Efficacy

The efficacy of a single intranasal immunization of hamsters with F-F3 strain was assessed in two independent experiments according to the scheme (Figure 1). In the first experiment, immunized hamsters were infected with Omicron-like FEB2 (BA.5.2-like) and Otradnoe (BA.1.1-like) strains and in the second experiment with FEB2 (BA.5.2-like) and Dubrovka (Wuhan-like) strains.

#### 3.4.1. Experiment 1

**Figure 5:**
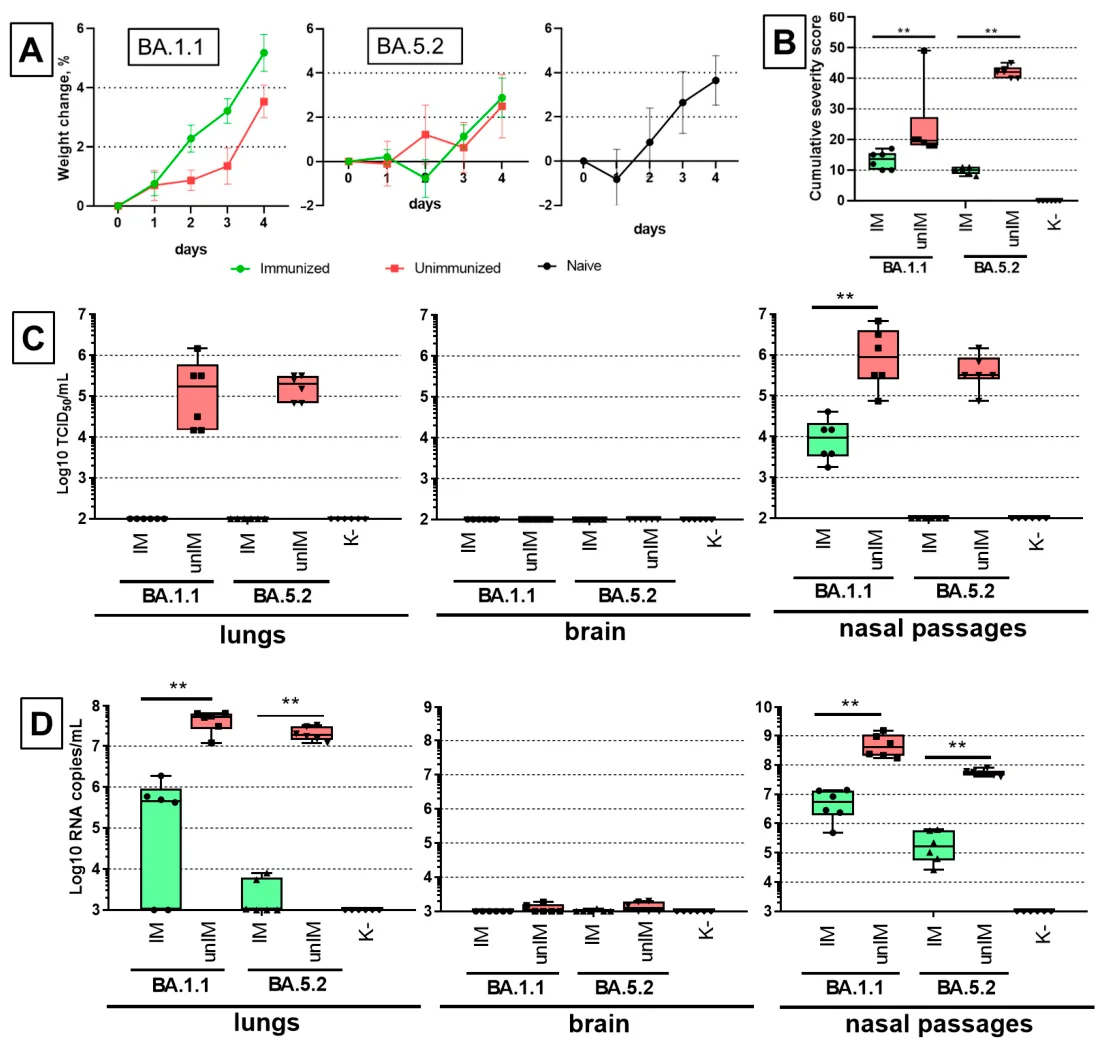
F-F3 strain efficacy in the COVID-19 Syrian hamster model (Experiment 1). Body weight changes in hamsters on days 1–4 p.c. (**A**); cumulative pneumonia severity score (**B**); virus titer (**C**) and viral RNA concentration (**D**) in lungs, brain, and nasal passages homogenates. **IM**—immunized hamsters; **unIM**—non-immunized hamsters; **K-**—non-immunized, non-challenged (naïve) hamsters. **BA.1.1**—challenge with the BA.1.1-like Otradnoe strain; **BA.5.2**—challenge with the BA.5.2-like FEB2 strain. **—*p* < 0.01 (Mann–Whitney U test). Data presented using individual data points and median/IQR.

No significant difference in the percentage change in body weight from baseline (day 0) to day 4 p.c. was observed between immunized and non-immunized animals following challenge with either the BA.5.2-like FEB2 or BA.1.1-like Otradnoe strain (Figure 5A). The absence of protection against weight loss is explained by the fact that the FEB2 and Otradnoe strains do not substantially retard weight gain in hamsters [34]. A more informative indicator of immunization efficacy was the viral load in the lungs and nasal passages. On day 4 post-challenge with the homologous BA.5.2-like strain, infectious virus was undetectable in the lungs and nasal passages of immunized hamsters. Following heterologous challenge with the BA.1.1-like strain, infectious virus was also undetectable in the lungs of immunized hamsters, whereas in the nasal passages, median virus titer was 3.9 log_10_ TCID_50_/mL, which was 2.0 log_10_ (95% CI: 1.3–2.9; *p* < 0.01) lower than that in the non-immunized control group. In all non-immunized hamsters, infectious virus was detected in the lungs and nasal passages at median titers 5.1 and 5.9 log_10_ TCID_50_/mL. Infectious virus was not detected in the brain of any animal group (Figure 5C).

Assessment of viral RNA content in the lungs and nasal passages confirmed the protective effect of immunization. Following challenge with the BA.5.2-like strain, viral RNA levels in the lungs and nasal passages of immunized animals were 4.2 log_10_ (95% CI: 3.4–4.5; *p* < 0.01) and 2.6 log_10_ (95% CI: 1.9–3.2; *p* < 0.01) lower, respectively, while after challenge with the BA.1.1-like strain, they were 2.0 log_10_ (95% CI: 1.5–4.7; *p* < 0.01) and 1.9 log_10_ (95% CI: 1.3–2.7; *p* < 0.01) lower compared to non-immunized animals. In the brain, viral RNA was detected only in individual non-immunized animals at the limit of detection (Figure 5D). Determination of viral RNA in other organs and tissues (heart, kidney, liver, spleen, blood) proved to be of limited utility for assessing immunization efficacy (Supplemental Figure S1).

At day 4 post-challenge with the homologous BA.5.2-like and heterologous BA.1.1-like strains, non-immunized hamsters developed bronchointerstitial pneumonia, with the most pronounced morphological manifestations of the inflammatory process observed following BA.5.2-like strain challenge. Lung morphology in hamsters from the intact control group (K-) corresponded to normal. Histological examination of the lungs of immunized hamsters at day 4 post-challenge with both strains revealed a marked reduction in the severity and extent of interstitial pneumonia. In lung sections from hamsters in these groups, small areas of reduced aeration corresponding to interstitial pneumonia foci were observed. Inflammatory changes in bronchi walls, bronchioles, and vessels were absent. Morphological signs of diffuse alveolar damage were not detected (Supplemental Figures S3 and S4). Blinded semi-quantitative scoring demonstrated significantly less severe pathomorphological changes in the lungs of immunized animals compared to non-immunized hamsters (Figure 5B, Supplemental Table S4). Specifically, the cumulative pneumonia severity score in immunized hamsters was reduced by 32 points (95% CI: 30–34; *p* < 0.01), corresponding to 76.2%, after homologous challenge and by 7.5 points (95% CI: 3–32; *p* < 0.01), corresponding to 30.8%, after heterologous challenge, relative to non-immunized controls.

#### 3.4.2. Experiment 2

**Figure 6:**
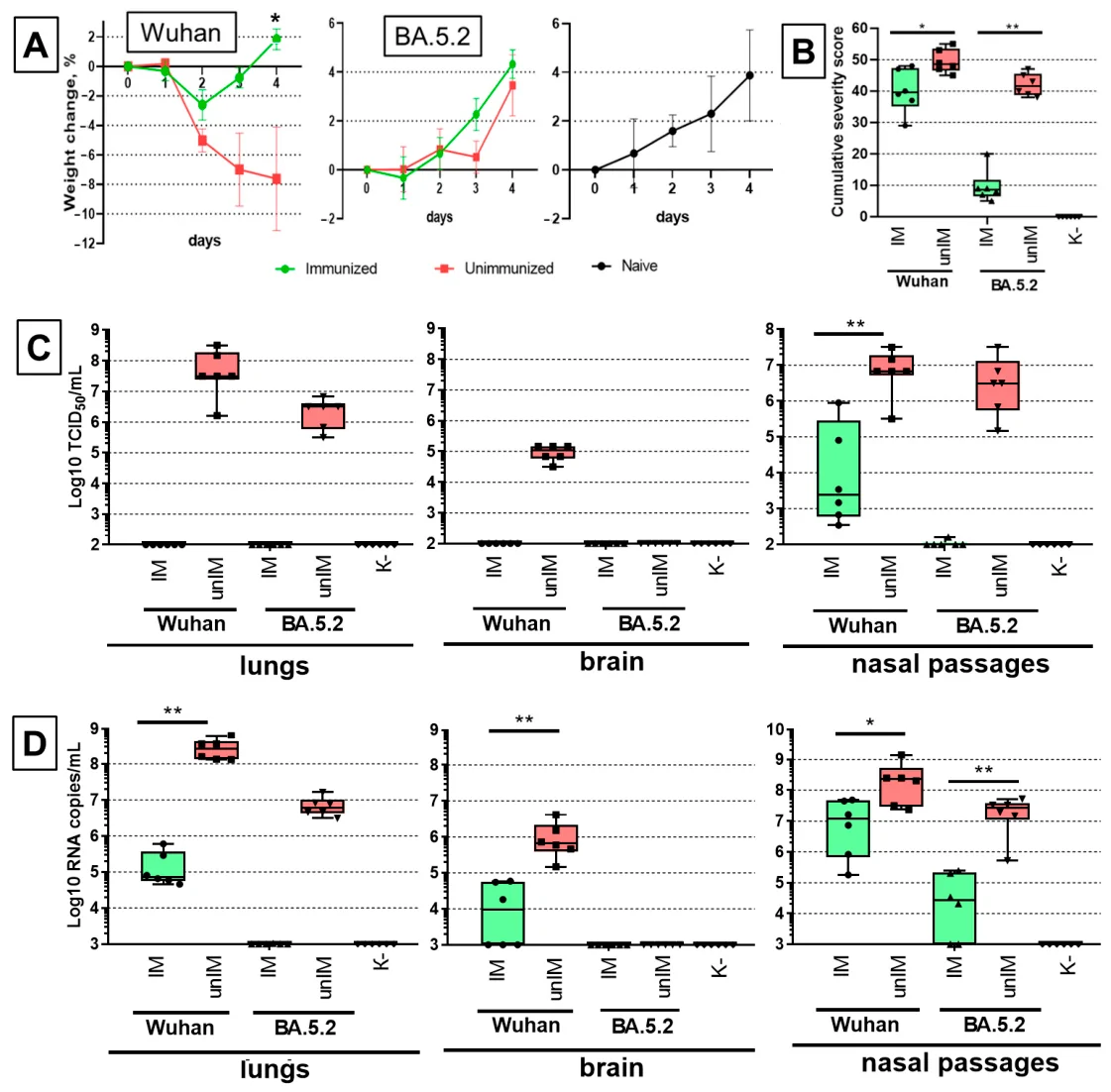
Efficacy of F-F3 strain in the COVID-19 Syrian hamster model (Experiment 2). Body weight changes in hamsters on days 1–4 p.c. (**A**); cumulative pneumonia severity score (**B**); virus titer (**C**) and viral RNA concentration (**D**) in lungs, brain, and nasal passages homogenates. **IM**—immunized hamsters; **unIM**—non-immunized hamsters; **K-**—non-immunized, non-challenged (naïve) hamsters. **Wuhan**—challenge with the Wuhan-like Dubrovka strain; **BA.5.2**—challenge with the BA.5.2-like FEB2 strain. **—*p* < 0.01, *—*p* < 0.05 (Mann–Whitney U test). Data presented using individual data points and median/IQR.

Following homologous challenge with the BA.5.2-like FEB2 strain, no significant difference in the percentage change in body weight on day 4 p.c. was observed between immunized and non-immunized animals. In contrast, following challenge with the highly virulent Wuhan-like Dubrovka strain, immunized animals showed a 13.5% (95% CI: −16.6 to −0.6; *p* < 0.05) smaller reduction in body weight by day 4 p.c. compared with non-immunized animals (Figure 6A).

At day 4 post-challenge with the BA.5.2-like and Wuhan-like strains, infectious virus was undetectable in the lungs of immunized hamsters, whereas in non-immunized animals, the median virus titers in the lungs were 6.3 and 7.2 log_10_ TCID_50_/mL, respectively. Following homologous challenge, infectious virus was not detected in the nasal passages of the majority of immunized hamsters (virus detected in only one of six animals at a titer of 2.2 log_10_ TCID_50_/mL), while all non-immunized animals had high titers of infectious virus (median 6.3 log_10_ TCID_50_/mL). Following heterologous challenge, infectious virus was recovered from the nasal passages of all immunized hamsters (median 3.4 log_10_ TCID_50_/mL), which was 3.3 log_10_ (95% CI: 1.2–4.3; *p* < 0.01) lower than in non-immunized animals. Upon challenge with the Wuhan-like strain, infectious virus was not detected in the brains of immunized hamsters, whereas it was detected in all non-immunized animals at a median titer of 5.0 log_10_ TCID_50_/mL (Figure 6C).

The results of viral RNA quantification in the lungs, nasal passages, and brain were well correlated with the data on infectious virus titers. The lungs of immunized hamsters were completely protected, while the nasal passages were partially protected against homologous challenge. Viral RNA was detected in the nasal passages, but its concentration was 2.8 log_10_ (95% CI: 1.9–4.5; *p* < 0.01) lower than in non-immunized animals. Following heterologous challenge, viral RNA levels in the lungs, brain, and nasal passages of immunized hamsters were 3.4 log_10_ (95% CI: 2.7–3.9; *p* < 0.01), 2.2 log_10_ (95% CI: 1.0–3.2; *p* < 0.01), and 1.4 log_10_ (95% CI: 0.3–2.5; *p* < 0.05) lower, respectively, than in non-immunized animals (Figure 6D). As in the first experiment, determination of viral RNA in other organs and tissues (heart, kidney, liver, spleen, blood) proved to be of limited utility for assessing immunization efficacy (Supplemental Figure S2).

Histological examination at day 4 post-challenge with the BA.5.2-like and Wuhan-like strains revealed bronchointerstitial pneumonia in the lungs of non-immunized hamsters, with inflammatory lesions involving 70–80% of the histological section area in some lobes. Lung histostructure in non-immunized, non-challenged hamsters (K- group) was normal. In the lungs of immunized hamsters, a marked reduction in the severity and extent of interstitial pneumonia was observed following homologous challenge with the BA.5.2-like strain. Airless areas were absent. In most microscopic fields, inflammatory infiltration of the interalveolar septa was moderate, with no exudate detected in the alveolar lumina. Peribronchiolar and perivascular lymphoid-histiocytic infiltrates were mild. Inflammatory changes in the bronchiolar walls were focal and mild. The epithelial lining of the conducting airways was preserved throughout. Vascular walls showed no pathological alterations. Following heterologous challenge, the severity of inflammatory changes was also reduced, although to a lesser extent. In some lobes, the area of pneumonic involvement reached 40–50%, whereas in other lobes, inflammatory changes were minimal. Pneumonic foci were predominantly localized along the major intrapulmonary airways and were absent in the peripheral regions. Overall, the severity of inflammatory changes in the bronchiolar walls, vessels, and alveoli was lower than in the non-immunized group (Supplemental Figures S5–S8). Blinded semi-quantitative scoring demonstrated a reduction in the cumulative pneumonia severity score of 33 points (95% CI: 27–38; *p* < 0.01), corresponding to 79.5%, in immunized hamsters compared with non-immunized animals after homologous challenge. After heterologous challenge, a more modest reduction of 8.5 points (95% CI: 1–18; *p* < 0.05), corresponding to 18.6%, was observed in immunized hamsters relative to non-immunized controls. (Figure 6B, Supplemental Table S4).

**Figure 7:**
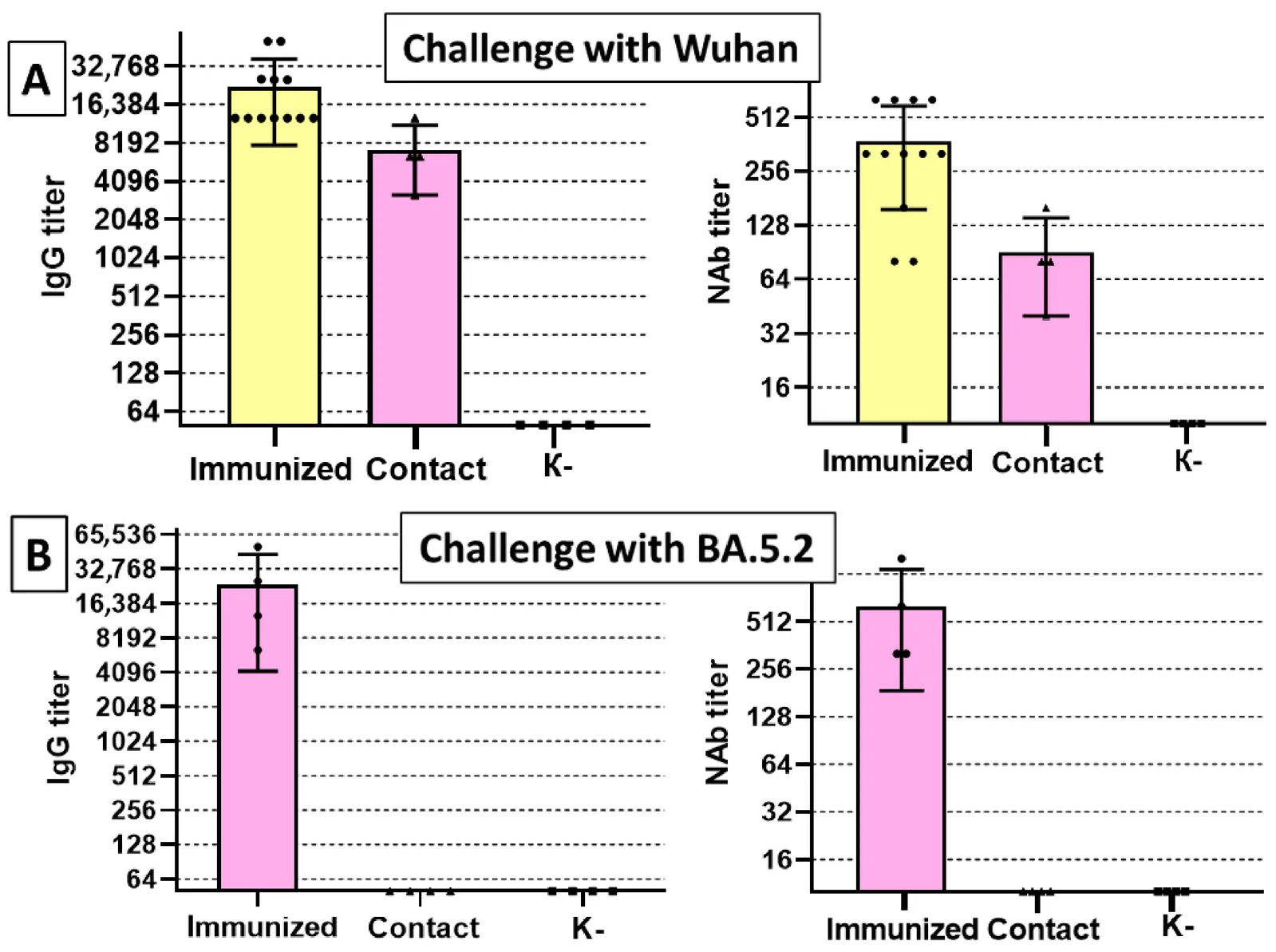
Protection of naïve contact hamsters from infection upon challenge of immunized animals with a virulent strain. Antibody titers to SARS-CoV-2 following heterologous challenge (21 days p.c.) of immunized hamsters with the Wuhan-like strain (**A**) and homologous challenge with the BA.5.2-like strain (**B**). NAb titer was determined against the challenge virus. **NAb**—neutralizing antibodies, **Contact**—co-housed naïve hamsters, **K-**—naïve animals. Error bars = SEM.

In addition, protection against transmission of a virulent strain to naïve co-housed hamsters following challenge of immunized animals was evaluated. When immunized hamsters were challenged with the heterologous Wuhan-like strain, co-housed hamsters were found to have total IgG antibodies to SARS-CoV-2 at 21 days p.c., indicating that they had become infected (Figure 7A). In contrast, when immunized hamsters were challenged with the homologous BA.5.2-like strain, co-housed hamsters were protected from infection, as evidenced by the absence of seroconversion at 21 days p.c. (Figure 7B). The presence or absence of antibody responses to SARS-CoV-2 was confirmed by virus neutralization assay (Figure 7A,B).

### 3.5. F-F3 Strain Transmissibility

**Figure 8:**
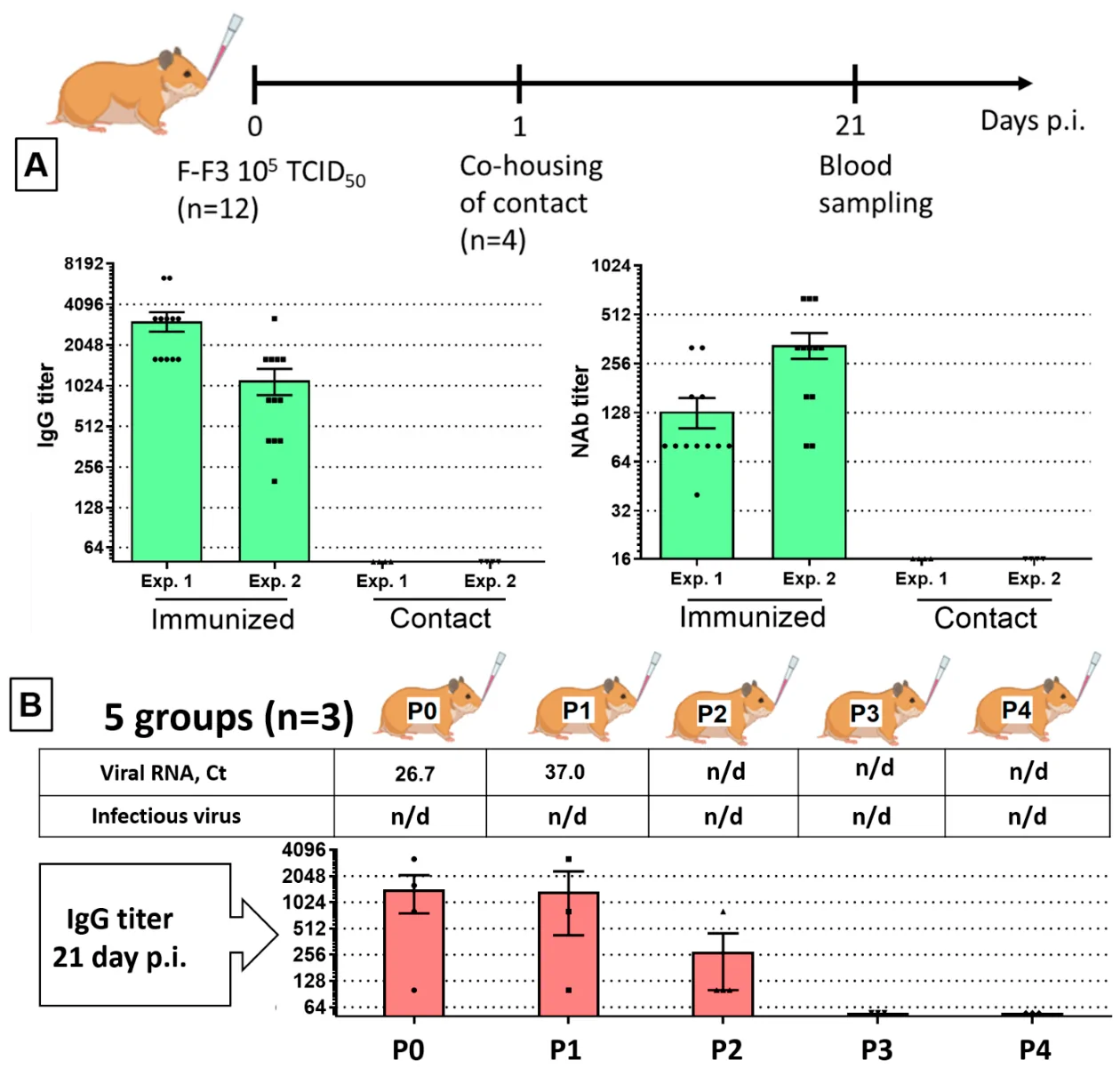
Assessment of F-F3 strain transmissibility. (**A**) Evaluation of transmissibility under co-housing of immunized and naïve hamsters. Titers of total and neutralizing antibodies in sera of immunized (*n* = 12) and co-housed (*n* = 4) hamsters at 21 days p.i. Data from two independent experiments. **Exp. 1**, **Exp. 2**—Experiment 1, Experiment 2. (**B**) Assessment of transmissibility by serial passaging of F-F3 strain in hamsters. **Contact**—co-housed naïve hamsters. **n/d**—not detected. Error bars = SEM.

All hamsters immunized with F-F3 strain developed serum IgG antibodies to SARS-CoV-2 structural antigens and neutralizing antibodies by day 21 p.i. In contrast, no seroconversion was detected in the four naïve contact hamsters co-housed with the immunized animals, as assessed by both ELISA and neutralization assay at day 21 p.i. (Figure 8A). A repeat experiment yielded identical results. Thus, in two independent experiments under the conditions tested, no natural transmission of the F-F3 strain to susceptible contact animals was detected, despite close proximity to immunized hamsters. Subsequently, an attempt was made to passage F-F3 strain in hamsters through sequential intranasal inoculation with nasal wash supernatants obtained from infected animals. IgG antibodies to SARS-CoV-2 were detected at day 21 p.i. in primarily infected hamsters (group P0) as well as after the first and second passages (groups P1 and P2), although antibody titers tended to decrease with increasing passage number, eventually falling below the detection limit. In P0 and P1 groups, low levels of viral RNA in nasal washes at day 3 p.i. were detected (Figure 8B), whereas in groups P2–P4, no viral RNA was detectable in the nasal passages. Notably, infectious virus was not detected by culture in nasal wash samples from any of the five groups at day 3 p.i. These findings showed that natural transmission was not detected in the co-housing experiments and that serial transfer of infectivity rapidly declined under the tested passage conditions.

## 4. Discussion

Among the approaches employed for SARS-CoV-2 attenuation, the generation of temperature-sensitive (*ts*) mutants is of particular interest. Sensitivity to 37 °C and above restricts viral replication to peripheral sites of the body, such as the epithelial cells of the upper respiratory tract, while precluding infection of internal organs. Consequently, SARS-CoV-2 *ts* mutant does not infect the lungs or induce pneumonia yet retains immunogenicity due to its ability to replicate in the upper respiratory tract. Using this approach, we previously demonstrated that the attenuated Wuhan-like *ts* D-D2 SARS-CoV-2 strain conferred highly effective protection in hamsters following a single intranasal immunization, not only against homologous but also against heterologous challenge, including Omicron-like strains [19]. In the present study, using the COVID-19 Syrian hamster model, we investigated for the first time the efficacy and safety of a prototype live attenuated vaccine based on a cold-adapted Omicron-like *ts* mutant (F-F3 strain).

Comparative analysis of all six *ca* mutant clones genomes derived from the FEB2 strain (including F-F3) does not fully elucidate the F-F3 attenuation mechanism, but it does provide insight into its genetic basis. The identified amino acid substitutions can be categorized into those common to all six *ca* mutants and those specific to individual clones (highlighted in different colors in Supplemental Table S3). Amino acid substitutions shared by all six *ca* mutants may be responsible for the novel properties acquired by all clones, such as adaptation to a new host (Vero monkey cells), the *ca* phenotype (ability to replicate at 24 °C), and sensitivity at 39 °C (inability to replicate at 39 °C). Furthermore, the *ca* mutants can be divided into those sensitive to 37 °C (F-F1, F-F3, F-D3, F-D12) and those insensitive to 37 °C (F-C5 and F-C9). Since temperature sensitivity is a critical attenuation marker in cold-adapted viruses, mutations that distinguish these two groups of clones are of particular interest for understanding the attenuation genetic determinants (underlined in Supplemental Table S3). Such substitutions in 37 °C-sensitive *ca* mutants were identified in the ORF1ab nsp13 domain (G5892C) in F-F1, F-F3, and F-D3 clones, and, at an adjacent position (G5891D) in F-D12 clone. In addition, F360L substitution in the N protein, present in the 37 °C-insensitive F-C5 and F-C9 clones and absent in all 37 °C-sensitive clones, is noteworthy. Thus, amino acid substitutions at positions 5891–5892 in the nsp13 domain of ORF1ab and at position 360 in N protein may play an important role in the F-F3 strain acquiring sensitivity to 37 °C. Furthermore, several other substitutions were identified in some 37 °C-sensitive clones that were not found in F-C5 and F-C9 clones and could theoretically contribute to their *ts* phenotype, in the nsp2 (V4578L) and nsp10 (K292N and V4578L) domains of ORF1ab, as well as in the structural M (A63T) and N (Q280K) proteins. However, the role of these substitutions in conferring sensitivity at 37 °C appears less likely, as they occur only in individual 37 °C-sensitive clones rather than in all of them. Importantly, without reverse-genetics experiments introducing or reverting these mutations individually or in combination, causality cannot be established.

Phenotypically, F-F3 strain is characterized *in vitro* by attenuation markers that distinguish it from the parental FEB2 strain, including the *ca* and *ts* phenotype. *In vivo*, F-F3 *att* phenotype is manifested by reduced immunogenicity (Figure 4) and the inability to infect hamsters’ lungs [35]. It is important to note that LAV safety is determined not only by the degree of vaccine strain attenuation but also by the *att* phenotype stability. F-F3 strain sensitivity to 37 °C underlies its inability to infect hamster lungs and induce pneumonia, thus representing a critical marker of viral attenuation. To assess F-F3 strain *ts* phenotype stability, we passaged it at 33 °C in Vero cells, which models replication of the vaccine strain in the upper respiratory tract of humans. After 10 passages at 33 °C, F-F3 strain retained its sensitivity to 37 °C and above, indicating *ts* phenotype stability (Figure 3). The unsuccessful attempt to recover infectious virus through serial “blind” passages in cell culture at 37 °C (Figure 3A) further confirmed F-F3 strain *ts* phenotype stability.

Following a single intranasal immunization with F-F3 strain, all hamsters developed IgG antibodies to SARS-CoV-2 that exhibited neutralizing activity against the homologous BA.5.2-like strain but did not neutralize the heterologous Wuhan-like strain (Figure 4). The use of both homologous BA.5.2-like and heterologous Wuhan-like strains for challenge of immunized hamsters allowed us to assess F-F3 cross-protective potential. Challenge with the Wuhan-like strain modeled a scenario in which an immunized population is exposed to a novel highly virulent SARS-CoV-2 strain with antigenic properties markedly different from those of the vaccine strain. Following a single intranasal administration, F-F3 strain reproducibly provided protection against bronchointerstitial pneumonia and productive infection in the lungs and nasal passages upon homologous challenge (Figure 5 and Figure 6). Upon heterologous challenge with the Wuhan-like virus, immunization did not prevent pneumonia but did reduce disease severity. So, it protected against weight loss and productive infection in the lungs and brain, as evidenced by significantly lower viral RNA concentrations in the lungs, nasal passages, and brain of immunized animals (Figure 6). Importantly, F-F3 immunization was efficacious against heterologous challenge despite the absence of detectable serum neutralizing activity against the Wuhan-like virus, suggesting a role for cellular immunity. However, as T-cell responses were not assessed in this study, this remains a hypothesis requiring experimental confirmation. Upon homologous challenge, immunization prevented transmission of the challenge virus to naïve contact hamsters co-housed with the experimental animals (Figure 7), in agreement with the findings of Lett M. et al. (2024) [20]. The lack of complete protection against heterologous challenge indicates the need for optimization of the immunization regimen. To enhance immunization efficacy, it appears reasonable to consider intranasal administration of a booster dose of a heterologous LAV or intramuscular immunization with an inactivated COVID-19 vaccine.

**Figure 9:**
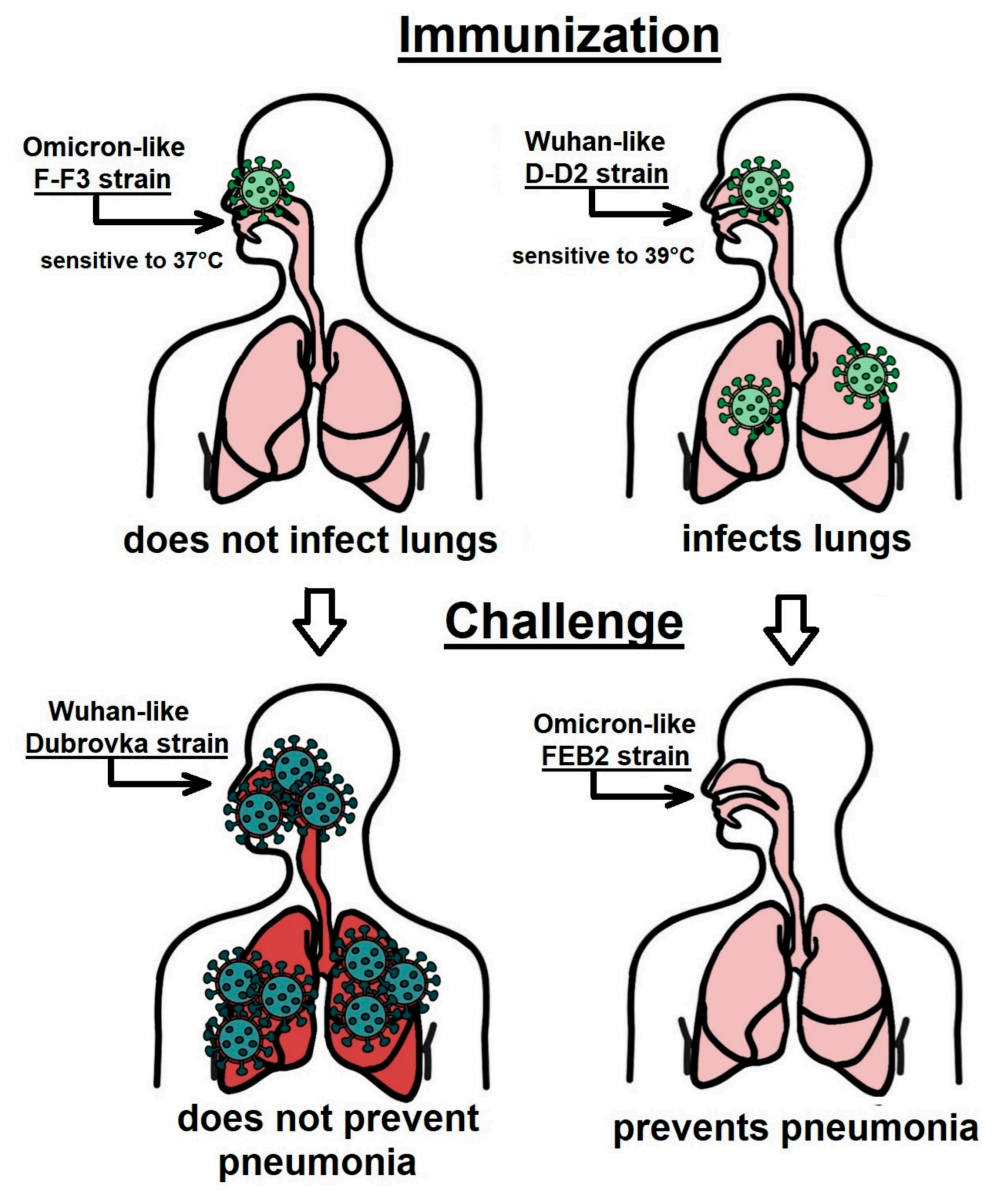
Protection against pneumonia upon **heterologous** challenge of hamsters immunized with SARS-CoV-2 *ts* mutants. The figure schematically illustrates the hypothetical relationship between the ability of a nasal LAV to protect against pneumonia upon **heterologous** challenge and the capacity of the vaccine strain to infect lungs. F-F3 strain (sensitive to 37 °C and above) does not infect lungs and does not protect against pneumonia, whereas D-D2 strain (sensitive to 39 °C and above) infects lungs and confers protection against pneumonia.

It is of interest to compare the efficacy of the Omicron-like F-F3 *ts* mutant with that of the Wuhan-like D-D2 *ts* mutant, which we previously characterized [19,28]. Both strains, F-F3 and D-D2, reproducibly protected hamsters from productive infection and pneumonia upon homologous challenge. However, upon heterologous challenge, D-D2 strain protected hamsters from pneumonia [19], whereas F-F3 strain did not. Notably, D-D2 strain exhibits sensitivity to 39 °C and above in cell culture but retains the ability to replicate at 37 °C, which underlies its limited capacity to infect hamster lungs upon immunization [19]. In contrast, F-F3 strain, which is sensitive to 37 °C and above, is unable to infect hamster lungs. These findings suggest that the ability of D-D2 strain to infect the lungs may account for its highly effective protection against pneumonia upon heterologous challenge (Figure 9). This can be explained by the activation of local immune mechanisms not only in nasopharyngeal mucosa (as is characteristic of F-F3 strain) but also in the lower respiratory tract (as is characteristic of D-D2 strain). However, the significant role of mucosal immune factors in protection against pneumonia upon heterologous challenge remains a hypothesis that requires experimental confirmation.

Licensed COVID-19 vaccines (including mRNA vaccines, viral vector-based, recombinant protein-based, and other platforms) are predominantly based on the SARS-CoV-2 S protein as the protective antigen, which exhibits the highest evolutionary variability [45]. The efficacy of such vaccines may decline upon the emergence of novel epidemic SARS-CoV-2 variants, a phenomenon most prominently observed in 2022, when the Omicron VOC achieved global predominance [46]. In 2022, the protection afforded by the first-generation COVID-19 mRNA vaccine against infection and symptomatic disease caused by Omicron-like strains was only 40–50% within the first 3 months p.i., followed by a rapid decline to 10–20% [47,48,49,50]. Moreover, Omicron sublineages BF.7, BQ.1, and XBB, which circulated in 2022–2023, were capable of escaping not only vaccine-induced immunity but also post-infection immunity acquired through prior infection with ancestral Omicron sublineages (BA.1, BA.2, etc.) [51]. Further evolution of Omicron descendants throughout 2024–2025 led to the emergence of novel variants, including LP.8.1.1, XEC.25.1, JN.1, NB.1.8.1, and XFG, which were characterized by antigenic changes associated with immune evasion from post-infection immunity [52,53,54]. To address this challenge, various approaches have been proposed, including periodic antigen updating of S-protein-based vaccines [55,56,57,58,59] and the development of a universal (pan-SARS-CoV-2) multi-epitope vaccine based on conserved B- and T-cell epitopes of protective viral antigens [60,61], among others. Taken together, our studies on prototype LAVs based on cold-adapted Wuhan-like [19] and Omicron-like (the present study) SARS-CoV-2 strains suggest that *ts* mutants could contribute to protection against emerging virus variants; however, this remains to be confirmed for contemporary circulating strains. Our findings are in good agreement with the data reported by Britzke T. et al. (2025) and Schön J. et al. (2024), who showed that the attenuated Wuhan-like OTS-228 strain induces sterilizing immunity upon homologous challenge and confers broad protection against Omicron-like genetic variants, including XBB.1.5 [62,63].

An attenuated vaccine strain, by replicating in respiratory epithelial cells, stably expresses the full repertoire of viral antigens. This enables the host to mount a robust and balanced immune response encompassing both humoral (neutralizing antibodies) and cellular (CD4^+^ and CD8^+^ T-cell activation) components, at both systemic and local (mucosal) levels [8,64,65]. Such a comprehensive response increases the likelihood of recognizing not only the homologous strain but also antigenically related heterologous strains. It is well established that anti-SARS-CoV-2 immunity involves not only the S protein but also other protective antigens carrying conserved T- and B-cell epitopes [66,67,68,69]. Indeed, the immunization efficacy of the recombinant N protein, as well as N protein encoded by mRNA, plasmid, or adenoviral vectors, has been convincingly demonstrated [70,71,72].

In addition to vaccine strain *ts* phenotype stability, an important safety parameter is its transmissibility. In the context of LAV clinical use, reversion to virulence of the vaccine strain could potentially occur as a result of transmission from immunized individuals to naïve contacts. In two independent experiments, we demonstrated that F-F3 transmission from immunized hamsters to naïve contact hamsters was not detected (Figure 8A), indicating that the likelihood of reversion to a virulent phenotype *in vivo* is minimized. Attempts to propagate F-F3 in hamsters through inoculation with nasal washes were also unsuccessful (Figure 8B), supporting the hypothesis that F-F3-immunized hamsters are non-contagious and reinforcing the absence of detectable natural transmission. However, we cannot characterize F-F3 as a replication-incompetent strain for hamsters, since infectious virus from nasal passages homogenates of all immunized animals was isolated in cell culture at 4 days p.i. [35]. The decline in seroconversion in P1 and P2 animals, progressing to the complete absence of anti-SARS-CoV-2 antibodies in P3 and P4 animals (Figure 8B), may result from low-level F-F3 replication in the hamster nasal passages or inefficient release of the virus into the nasal cavity, which together reduce the efficiency of virus transmission. A similar inability to passage the attenuated OTS-228 SARS-CoV-2 strain in hamsters using nasal washes was described by Britzke T. et al. (2025). In the absence of infectious virus in the nasal cavity lumen, the authors performed *in vivo* passaging of OTS-228 strain using nasal tissues homogenates [62]. An additional important F-F3 strain safety feature is its lack of neurovirulence for hamsters, which is genetically determined by the inability of the parental FEB2 strain to infect the brain [34]. Thus, the F-F3 strain possessed a stable *ts* phenotype and showed no evidence of transmission to hamsters under the conditions tested, which together support its safety profile.

**Limitations of the study.** Several limitations of the study concerning the Syrian hamster COVID-19 model should be acknowledged, in addition to the possible influence of the cage effect on the results mentioned in the “Statistical analysis” subsection. The first limitation is the difficulty of extrapolating results obtained in Syrian hamsters to humans, since the COVID-19 Syrian hamster model, although recapitulating mild-to-moderate, self-limiting disease, fails to reproduce severe or fatal outcomes, asymptomatic courses, extrapulmonary vascular lesions, or thromboses. Second, only four-week-old female hamsters were used, which precludes assessment of sex- and age-dependent differences in vaccine responses and protection. Third, most efficacy outcomes were evaluated at a single early time point, day 4 p.c., and therefore neither longer-term viral clearance nor the duration of protection could be assessed. Forth, it is important to note that, while the ancestral Wuhan-like challenge provides proof-of-concept for heterologous protection by the F-F3 strain, cross-protection against contemporary circulating Omicron lineages was not assessed, which limits the clinical relevance of these findings. Nevertheless, the Syrian hamster model has proven highly valuable, as it reproduces key features of human COVID-19, including pathogenesis and viral transmission, and allows assessment of vaccine efficacy. In addition, this study assessed only the phenotypic stability of F-F3 under the tested *in vitro* conditions, and the emergence of revertants or compensatory mutations under other conditions cannot be fully excluded. Finally, it should be noted that this study did not assess mucosal IgA or T cell responses, which limits our ability to fully interpret the mechanisms underlying the protection observed.

## 5. Conclusions

Preclinical studies of the prototype LAV based on the *ts* Omicron-like F-F3 strain have demonstrated a favorable balance between its safety and efficacy. The F-F3 strain does not infect hamster lungs or brain, shows no evidence of transmission to co-housed naïve animals under the conditions tested, and possesses a stable *ts* phenotype *in vitro*. At the same time, F-F3 confers strong protection against productive lung infection and marked reduction in pneumonia severity upon homologous challenge and partial protection against heterologous challenge. The use of the ancestral Wuhan strain for challenge demonstrated the protective potential of F-F3 against a phylogenetically distant, highly virulent strain. The complex of the F-F3 strain characteristics supports its consideration as a candidate strain for the development of a COVID-19 LAV. Furthermore, the F-F3 strain represents a promising potential “donor of attenuation” for design of LAV, with the possibility of optimizing the antigenic properties of the S protein through genetic engineering, as demonstrated in the study by Kochmann J. et al. (2026) [73].

## Figures and Tables

**Table 1:** SARS-CoV-2 strains used in the study.

Strain Name	GenBank ID	Pango Lineage	Replication Capacity at Temperature			Ability to Infect Lungs	Source, Date, and Place of Isolation	Passage History in Vero Cells	Reference
			24 °C	37 °C	39 °C				
Dubrovka	MW514307.1	B.1.1.317	no	yes	yes	yes	COVID-19 patient, June 2020, Moscow, Russia	17 passages at 37 °C	[34]
Otradnoe	ON032857.1	BA.1.1	no	yes	yes	yes	COVID-19 patient, January 2022, Moscow, Russia	8 passages at 37 °C	
FEB2	OP920753.1	BA.5.2	no	yes	yes	yes	COVID-19 patient, October 2022, Moscow, Russia	4 passages at 37 °C	
F-F1	PX401969.1	BA.5.2	yes	no	no	n/a	Cold-adapted FEB2 strain mutants	27 passages at a temperature gradually decreasing from 37 °C to 24 °C	[35]
F-F3	PX401966.1	BA.5.2	yes	no	no	no			
F-D3	PX401970.1	BA.5.2	yes	no	no	n/a			
F-D12	PX401968.1	BA.5.2	yes	no	no	n/a			
F-C5	PX401967.1	BA.5.2	yes	yes	no	n/a			
F-C9	PX401971.1	BA.5.2	yes	yes	no	n/a			

Abbreviations: **n/a**—not analyzed.

## Data Availability

The genomic sequences of FEB2 strain *ca* mutants (F-F1, F-F3, F-D3, F-D12, F-C5, and F-C9) are available in the GenBank database (accession numbers PX401966–PX401971). The values of the virus titer and the concentration of viral RNA in the organs of individual animals analyzed in this study, as well as antibody titer and other raw data, will be made available by the authors on request.

## Supplementary Materials

The following supporting information can be downloaded at https://www.mdpi.com/article/10.3390/vaccines14100895/s1: Table S1: The distribution of animals across groups and cages for experiments involving animals; Table S2: Nucleotide substitutions acquired by FEB2 strain cold-adapted mutants following adaptation to Vero cells; Table S3: Amino acid substitutions acquired by FEB2 strain cold-adapted mutants following adaptation to Vero cells; Table S4: Individual cumulative pneumonia severity scores for efficacy experiments 1 and 2; Figure S1: Viral RNA concentration in homogenates of the heart, kidney, liver, spleen, and blood of immunized and non-immunized hamsters (Experiment 1); Figure S2: Viral RNA concentration in homogenates of the heart, kidney, liver, spleen, and blood of immunized and non-immunized hamsters (Experiment 2); Figure S3: Morphology of non-immunized hamster lungs at day 4 post-challenge with SARS-CoV-2 strains (Experiment 1); Figure S4: Morphology of immunized hamster lungs at day 4 post-challenge with SARS-CoV-2 strains (Experiment 1); Figure S5: Morphology of non-immunized hamster lungs at day 4 post-challenge with the Wuhan-like Dubrovka strain (Experiment 2); Figure S6: Morphology of non-immunized hamster lungs at day 4 post-challenge with the BA.5.2-like FEB2 strain (Experiment 2); Figure S7: Morphology of immunized hamster lungs at day 4 post-challenge with the Wuhan-like Dubrovka strain (Experiment 2); Figure S8: Morphology of immunized hamster lungs at day 4 post-challenge with the BA.5.2-like FEB2 strain (Experiment 2).

## Abbreviations

The following abbreviations are used in this manuscript:

*att*	Attenuated
*ca*	Cold-adapted
IQR	Interquartile range
LAV	Live attenuated vaccine
MOI	Multiplicity of infection
NAb	Neutralizing antibodies
p.c.	Post-challenge
p.i.	Post-infection or post-immunization (depending on the context)
PBS	Phosphate-buffered saline
RT-PCR	Reverse transcription polymerase chain reaction
SEM	Standard error of the mean
TCID_50_	50% tissue culture infectious dose
*ts*	Temperature-sensitive
