# Supplementary material for: Efficacy and Safety of Live Attenuated COVID-19 Vaccine Candidate Based on Omicron-like Temperature-Sensitive Mutant in Preclinical Study
Source: Vaccines (Basel). 2026 Oct 13;14(10):895. doi: 10.3390/vaccines14100895 (PMC13709953; doi:10.3390/vaccines14100895)
Supplement: Supplementary file 1 [file vaccines-14-00895-s001.zip › vaccines-4588300-supplementary.pdf]

Supplement

Supplemental Table S1. The distribution of animals across groups and cages for experiments involving animals.

Experiment	Group	Number of animals	Cages	Animals per cage	Figure
Immunogenicity Exp. 1	Immunized F-F3	12	1	12	Fig. 4
Immunogenicity Exp. 1	Immunized FEB2	6	1	6	
Immunogenicity Exp. 1	Unimmunized (K-)	6	1	6	
Immunogenicity Exp. 2	Immunized F-F3	12	1	12	
Immunogenicity Exp. 2	Unimmunized (K-)	6	1	6	
Efficacy Exp. 1	Immunized + BA.1.1 challenge	6	1	6	Fig. 5
Efficacy Exp. 1	Immunized + BA.5.2 challenge	6	1	6	
Efficacy Exp. 1	Unimmunized + BA.1.1 challenge	6	1	6	
Efficacy Exp. 1	Unimmunized + BA.5.2 challenge	6	1	6	
Efficacy Exp. 1	Naïve animals (K-)	6	1	6	
Efficacy Exp. 2	Immunized + Wuhan challenge	6	1	6	Fig. 6
Efficacy Exp. 2	Immunized + BA.5.2 challenge	6	1	6	
Efficacy Exp. 2	Unimmunized + Wuhan challenge	6	1	6	
Efficacy Exp. 2	Unimmunized + BA.5.2 challenge	6	1	6	
Efficacy Exp. 2	Naïve animals (K-)	6	1	6	
Protection against transmission	Immunized + Wuhan challenge	12	1	16	Fig. 7
Protection against transmission	Contact (Wuhan challenge)	4			
Protection against transmission	K-	4	1	4	
Protection against transmission	Immunized + BA.5.2 challenge	4	1	8	
Protection against transmission	Contact (BA.5.2 challenge)	4			
Protection against transmission	K-	4	1	4	
Transmissibility Exp. 1	Immunized	12	1	16	Fig. 8
Transmissibility Exp. 1	Naïve contacts	4			
Transmissibility Exp. 2	Immunized	12	1	16	
Transmissibility Exp. 2	Naïve contacts	4			

Supplemental Table S2. Nucleotide substitutions acquired by FEB2 strain cold-adapted mutants following adaptation to Vero cells.

<table>
<tr><th rowspan="2">Clone</th><th rowspan="2">5'-UTR</th><th colspan="10">ORF1ab</th></tr>
<tr><th>nsp2</th><th>nsp3</th><th>nsp4</th><th>nsp5</th><th>nsp6</th><th>nsp10</th><th>nsp12</th><th>nsp13</th><th>nsp14</th><th>nsp15</th></tr>
<tr><td>F-C9</td><td>—</td><td rowspan="3">—</td><td rowspan="4">C3154T</td><td>—</td><td rowspan="6">C10175T
C10801T
C11422T</td><td rowspan="6">C13132T</td><td rowspan="2">—</td><td rowspan="2">T15353C G17227T</td><td>—</td><td rowspan="6">C19515T,
C19863T</td><td>C20072G</td></tr>
<tr><td>F-C5</td><td>N187G</td><td>G9117T</td><td>G17535A</td><td rowspan="5">—</td></tr>
<tr><td>F-D12</td><td>—</td><td rowspan="4">—</td><td>G13976T</td><td>T15353C</td><td>G17535A G17916A</td></tr>
<tr><td>F-D3</td><td>N187G</td><td rowspan="2">G1118T</td><td>—</td><td>T15353C G17227T</td><td>G17915T</td></tr>
<tr><td>F-F1</td><td>—</td><td>—</td><td>G13976T</td><td rowspan="2">T15353C</td><td>G17915T C19452G</td></tr>
<tr><td>F-F3</td><td>N187G</td><td>—</td><td>C3154T</td><td>—</td><td>G17535A G17915T</td></tr>
</table>

Supplemental Table S2 (continued).

<table>
<tr><th>Clone</th><th>S</th><th>E</th><th>M</th><th>ORF6</th><th>N</th></tr>
<tr><td>F-C9</td><td>G23754T, A24339T, C24348A, C24349G, C24756G</td><td rowspan="6">G26316A</td><td rowspan="4">G26488A, C26501T</td><td>—</td><td>G28507T, C28626A, C29312A</td></tr>
<tr><td>F-C5</td><td>G23754T, A24339T, C24348A, C24349G</td><td>G27325T</td><td>G28507T C28626A G28866A C29312A</td></tr>
<tr><td>F-D12</td><td>G23079C, G23754T, A24339T, C24348A, C24349G, C24756T</td><td>—</td><td rowspan="3">G28507T C28626A C29312T</td></tr>
<tr><td>F-D3</td><td>G23754T, A24339T, C24348A, C24349G</td><td>G27325T</td></tr>
<tr><td>F-F1</td><td>G21729A, G23079C, G23754T, A24339T, C24348A, C24349G</td><td rowspan="2">G26488A, C26501T, A26668G</td><td rowspan="2">—</td></tr>
<tr><td>F-F3</td><td>T22772G, G23754T, A24339T, C24348A, C24349G</td><td>G28507T C28626A C29070A C29312T</td></tr>
</table>

The coordinates are given according to FEB2 strain genome (GenBank ID OP920753.1).

Supplemental Table S3. Amino acid substitutions acquired by FEB2 strain cold-adapted mutants following adaptation to Vero cells.

<table>
<tr><th>Clone</th><th>nsp2</th><th>nsp3</th><th>nsp4</th><th>nsp5</th><th>nsp6</th><th>nsp10</th><th>nsp12</th><th>nsp13</th><th>nsp14</th><th>nsp15</th></tr>
<tr><td>C9</td><td rowspan="3">—</td><td rowspan="4">P971L</td><td>—</td><td rowspan="6">T3520I, A3727V</td><td rowspan="6">T4297I</td><td rowspan="2">—</td><td rowspan="2">S5038P, E5661D</td><td>—</td><td rowspan="6">S6425L, A6541V</td><td>L6610V</td></tr>
<tr><td>C5</td><td>G2959C</td><td>C5765Y</td><td rowspan="5">—</td></tr>
<tr><td>D12</td><td rowspan="4">—</td><td>V4578L</td><td>S5038P</td><td>C5765Y, <u>G5891D</u></td></tr>
<tr><td>D3</td><td rowspan="2">K292N</td><td></td><td>S5038P, E5661D</td><td><u>G5892C</u></td></tr>
<tr><td>F1</td><td>—</td><td>V4578L</td><td rowspan="2">S5038P</td><td><u>G5892C</u> A6403G</td></tr>
<tr><td>F3</td><td>—</td><td>P971L</td><td>—</td><td>C5765Y <u>G5892C</u></td></tr>
</table>

Supplemental Table S3 (continued).

<table>
<tr><th>Clone</th><th>S</th><th>E</th><th>M</th><th>ORF6</th><th>N</th></tr>
<tr><td>C9</td><td>D742Y, T937S, P940S, P1079A</td><td rowspan="6">R38Q</td><td rowspan="4">D3N, T7I</td><td>—</td><td>R92L, P132T, <u>F360L</u></td></tr>
<tr><td>C5</td><td>D742Y, T937S, P940S</td><td>E55D</td><td>R92L, P132T, G212S, <u>F360L</u></td></tr>
<tr><td>D12</td><td>A517P, D742Y, T937S, P940S, P1079S</td><td>—</td><td rowspan="3">R92L, P132T</td></tr>
<tr><td>D3</td><td>D742Y, T937S, P940S</td><td>E55D</td></tr>
<tr><td>F1</td><td>V70I, A517P, D742Y, T937S, P940S</td><td rowspan="2">D3N, T7I, A63T</td><td rowspan="2">—</td></tr>
<tr><td>F3</td><td>N414K, D742Y, T937S, P940S</td><td>R92L, P132T, Q280K</td></tr>
</table>

Amino acid substitutions highlighted in blue are common to all six clones. Amino acid differences between clones sensitive to 37°C (F-F1, F-F3, F-D3, F-D12) and those insensitive to 37°C (F-C5 and F-C9) are indicated in red. Substitutions that are presumed to play an important role in conferring sensitivity to 37°C are underlined. The coordinates are given according to FEB2 strain ORFs (GenBank ID OP920753.1).

Supplemental Table S4. Individual cumulative pneumonia severity scores for efficacy experiments 1 and 2.

**Experiment 1 (Figure 5B)**						
**Group**	**Individual cumulative pneumonia severity scores**					
Immunized + BA.1.1 challenge	10	10	17	15	12	15
Unimmunized + BA.1.1 challenge	18	19	49	20	18	20
Immunized + BA.5.2 challenge	11	8	11	10	9	10
Unimmunized + BA.5.2 challenge	40	42	45	40	42	43
Naïve animals (K-)	0	0	0	0	0	0
**Experiment 2 (Figure 6B)**						
Immunized + Wuhan challenge	40	29	39	48	37	47
Unimmunized + Wuhan challenge	55	53	45	49	47	48
Immunized + BA.5.2 challenge	20	8	5	7	9	9
Unimmunized + BA.5.2 challenge	39	47	40	38	45	43
Naïve animals (K-)	0	0	0	0	0	0

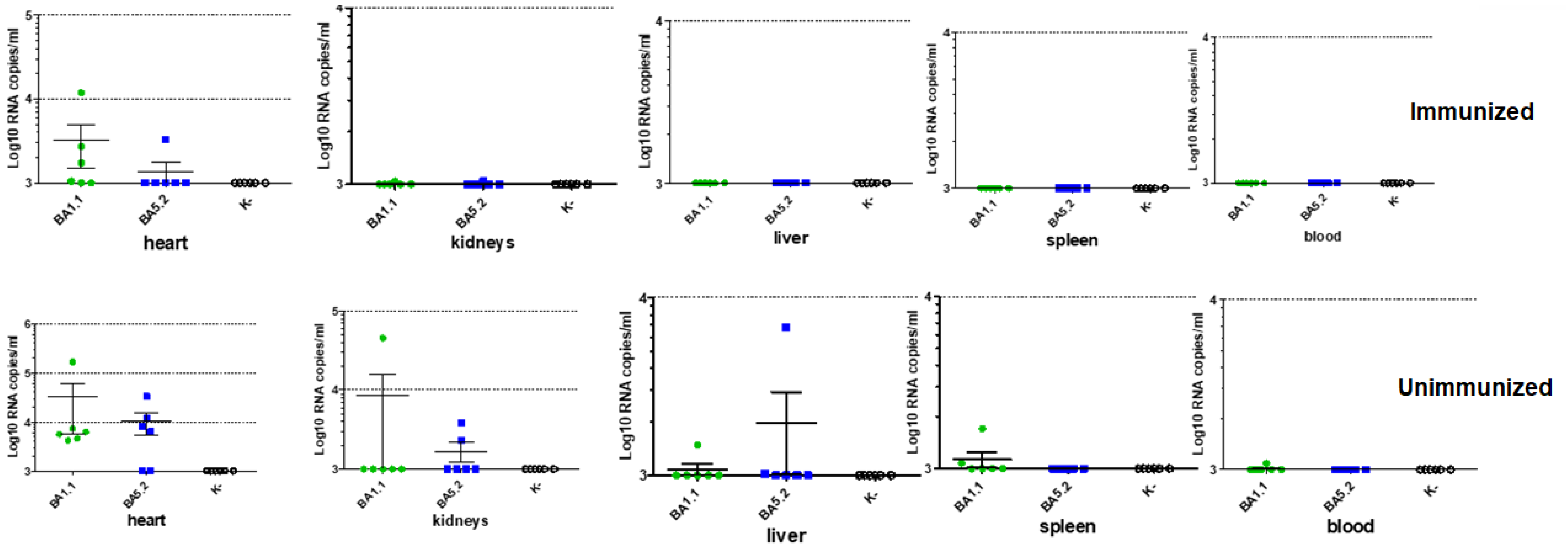

Supplemental Figure S1. Viral RNA concentration in homogenates of the heart, kidney, liver, spleen, and blood of immunized and non-immunized hamsters (Experiment 1).

BA.1.1 – challenge with the BA.1.1-like Otradnoe strain; **BA.5.2** – challenge with the BA.5.2-like FEB2 strain; **K–** – non-immunized, non-challenged (naïve) hamsters.

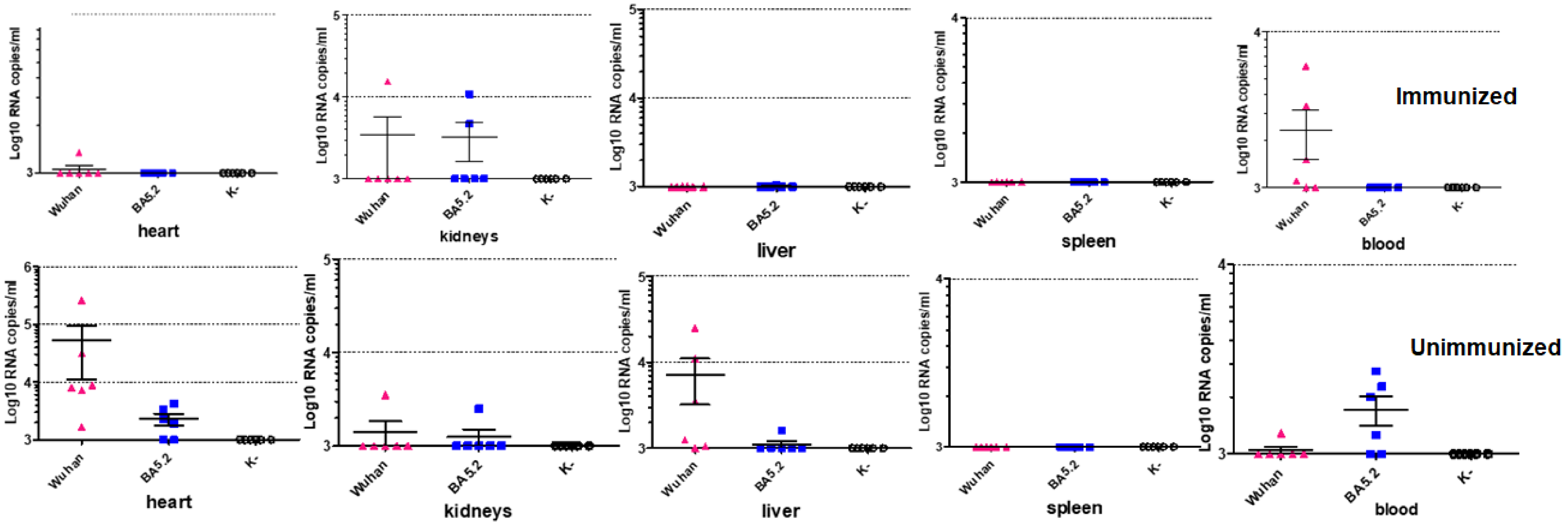

Supplemental Figure S2. Viral RNA concentration in homogenates of the heart, kidney, liver, spleen, and blood of immunized and non-immunized hamsters (Experiment 2).

Wuhan – challenge with the Wuhan-like Dubrovka strain; **BA.5.2** – challenge with the BA.5.2-like FEB2 strain; **K–** – non-immunized, non-challenged (naïve) hamsters.

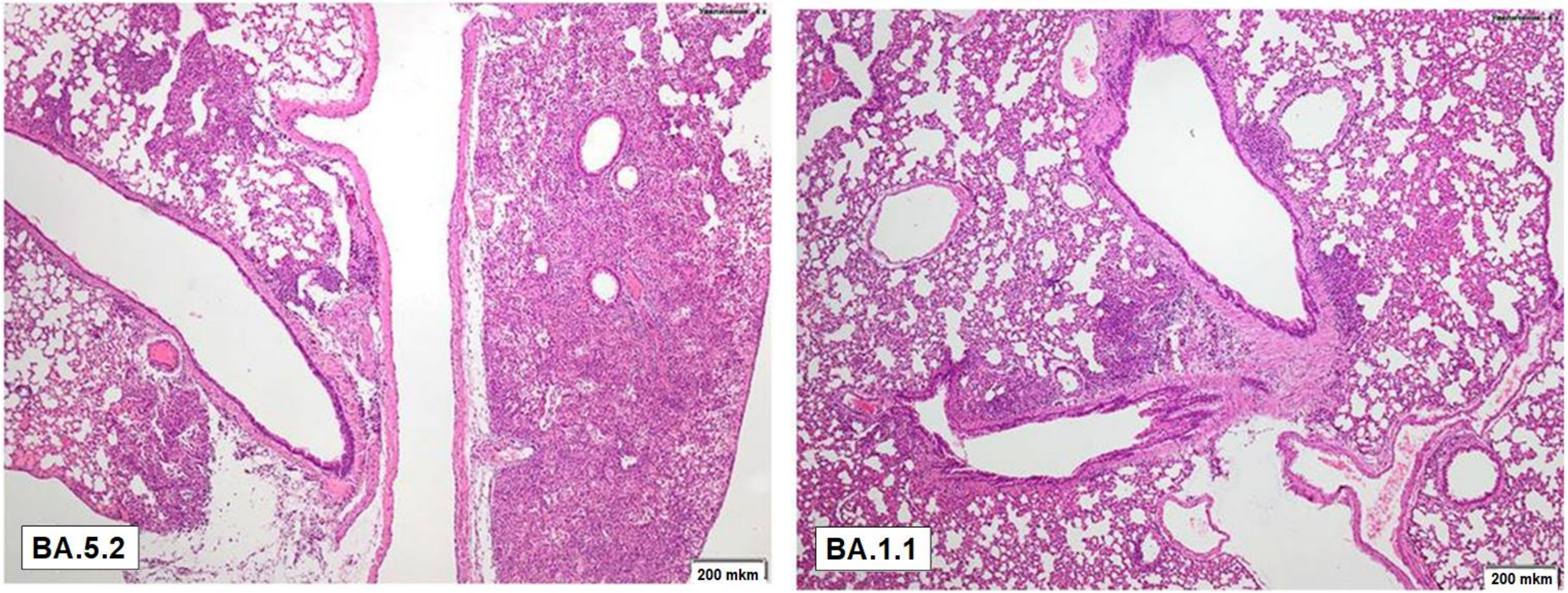

Supplemental Figure S3. Morphology of non-immunized hamster lungs at day 4 post-challenge with SARS-CoV-2 strains (Experiment 1).

BA.5.2 – challenge with the BA.5.2-like FEB2 strain; **BA.1.1** – challenge with the BA.1.1-like Otradnoe strain. Foci of interstitial and bronchointerstitial pneumonia in the lungs. Stained with hematoxylin and eosin.

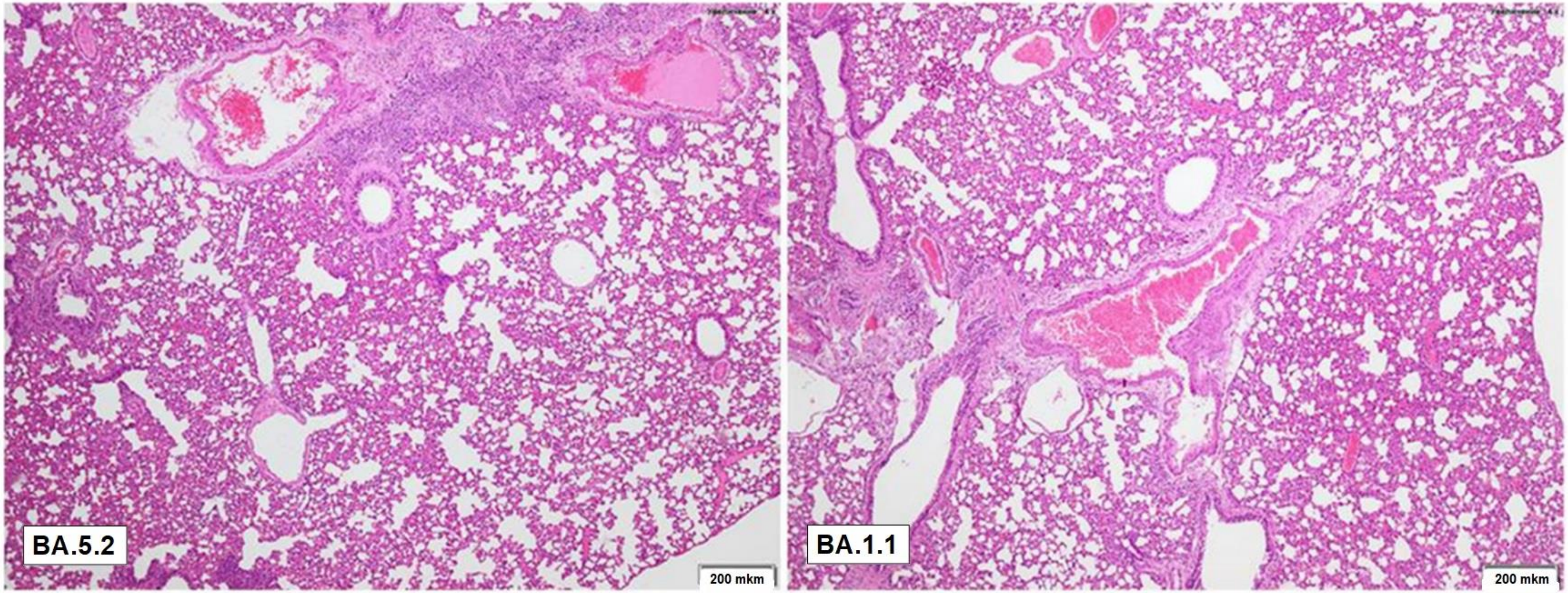

Supplemental Figure S4. Morphology of immunized hamster lungs at day 4 post-challenge with SARS-CoV-2 strains (Experiment 1).

BA.5.2 – challenge with the BA.5.2 like FEB2 strain; **BA.1.1** – challenge with the BA.1.1 like Otradnoe strain. Small foci of interstitial pneumonia. Stained with hematoxylin and eosin.

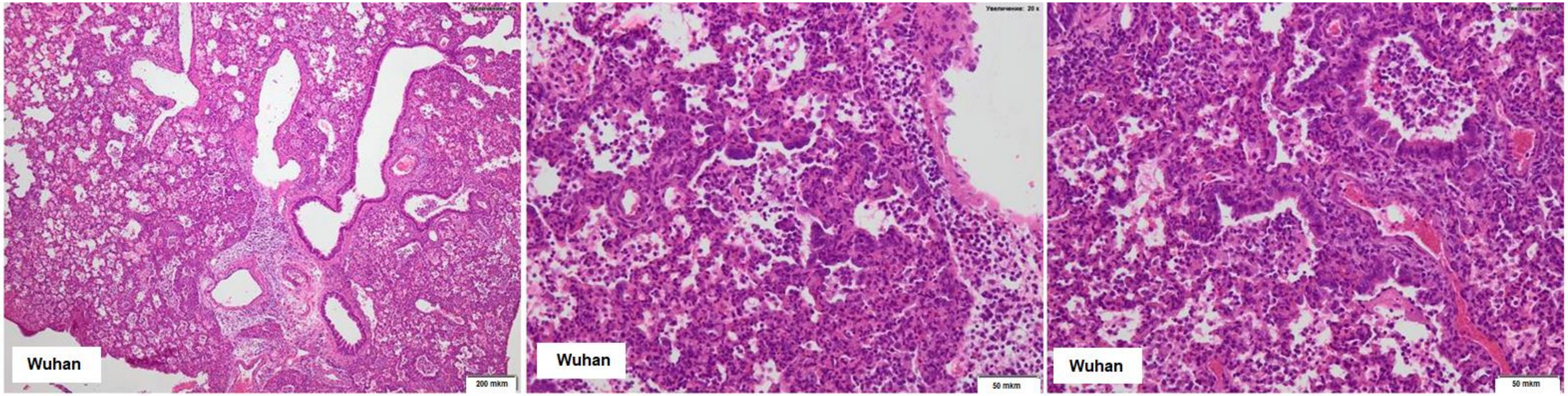

Supplemental Figure S5. Morphology of non-immunized hamster lungs at day 4 post-challenge with the Wuhan-like Dubrovka strain (Experiment 2). Bronchointerstitial pneumonia. Stained with hematoxylin and eosin.

Wuhan – challenge with the Wuhan-like Dubrovka strain.

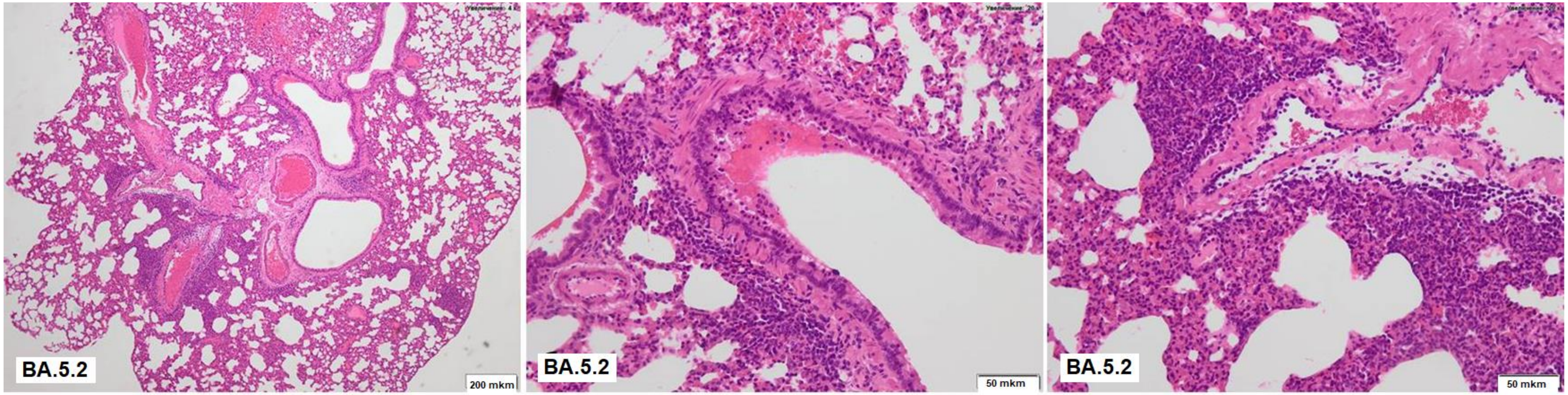

Supplemental Figure S6. Morphology of non-immunized hamster lungs at day 4 post-challenge with the BA.5.2-like FEB2 strain (Experiment 2). Bronchointerstitial pneumonia. Stained with hematoxylin and eosin.

BA.5.2 – challenge with the BA.5.2-like FEB2 strain.

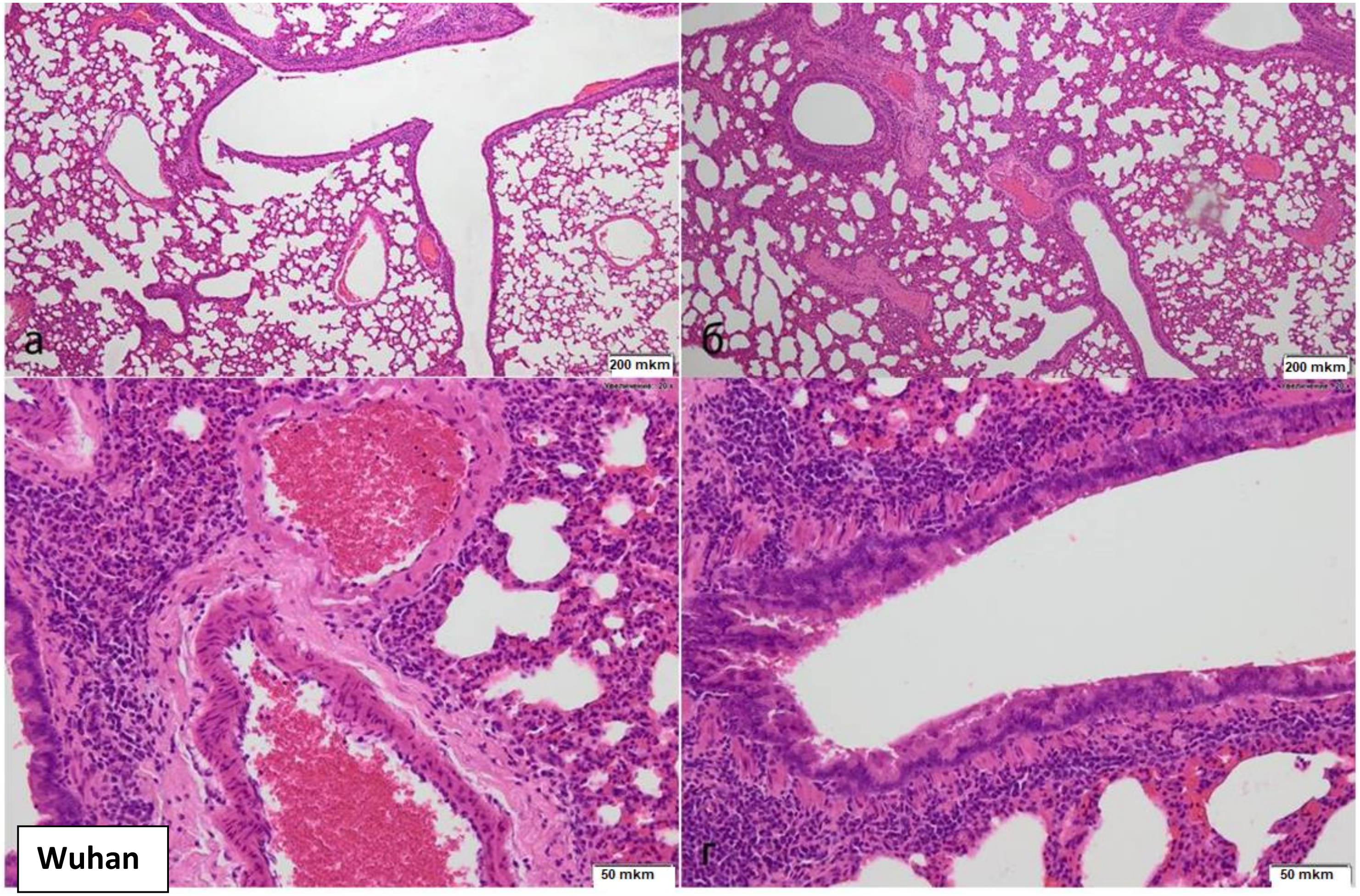

Supplemental Figure S7. Morphology of immunized hamster lungs at day 4 post-challenge with the Wuhan-like Dubrovka strain (Experiment 2). Bronchointerstitial pneumonia. Stained with hematoxylin and eosin.

Wuhan – challenge with the Wuhan-like Dubrovka strain.

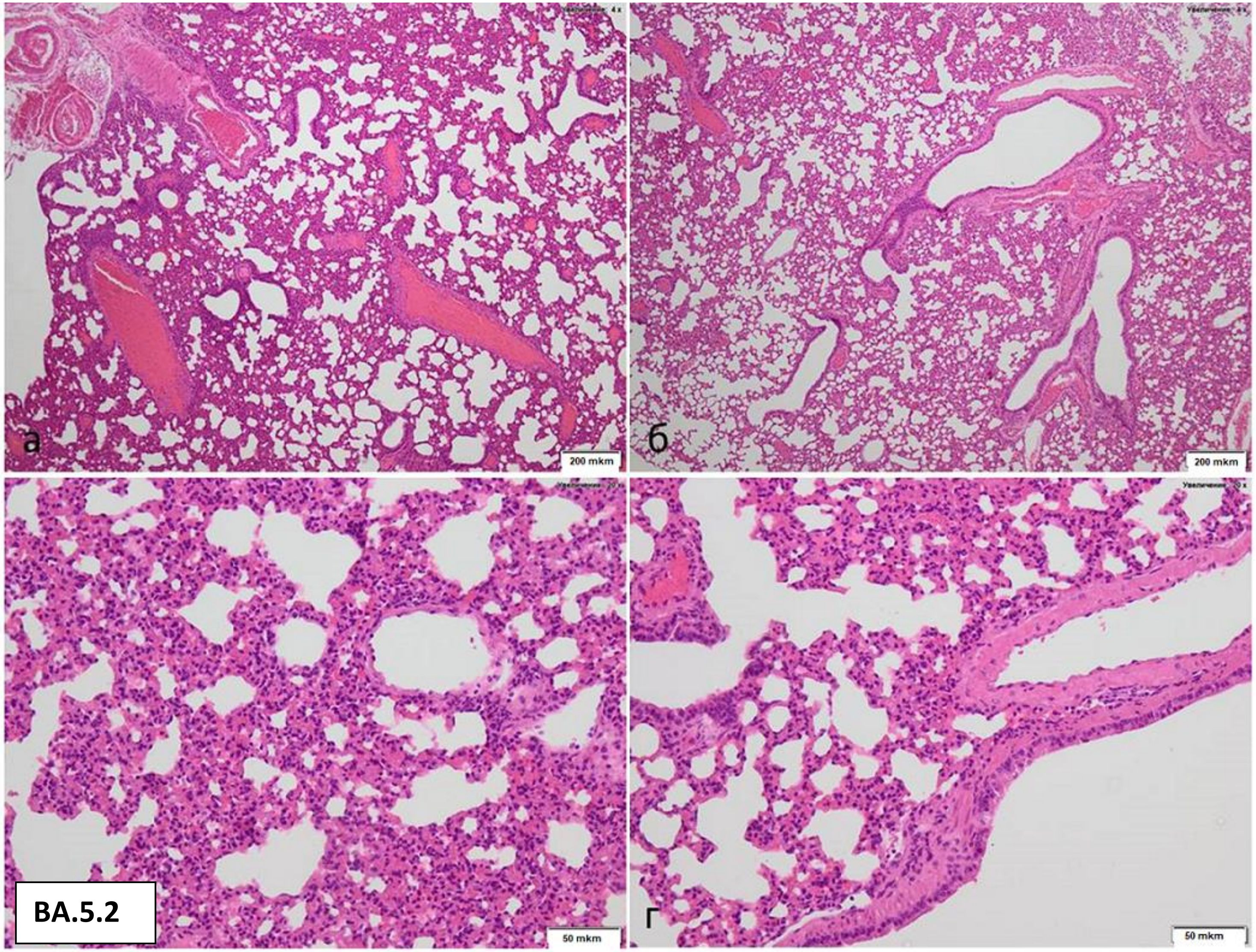

Supplemental Figure S8. Morphology of immunized hamster lungs at day 4 post-challenge with the BA.5.2-like FEB2 strain (Experiment 2). Mild interstitial pneumonia. Stained with hematoxylin and eosin.

BA.5.2 – challenge with the BA.5.2-like FEB2 strain.
